# Multi-level analysis of the gut–brain axis shows autism spectrum disorder-associated molecular and microbial profiles

**DOI:** 10.1038/s41593-023-01361-0

**Published:** 2023-06-26

**Authors:** James T. Morton, Dong-Min Jin, Robert H. Mills, Yan Shao, Gibraan Rahman, Daniel McDonald, Qiyun Zhu, Metin Balaban, Yueyu Jiang, Kalen Cantrell, Antonio Gonzalez, Julie Carmel, Linoy Mia Frankiensztajn, Sandra Martin-Brevet, Kirsten Berding, Brittany D. Needham, María Fernanda Zurita, Maude David, Olga V. Averina, Alexey S. Kovtun, Antonio Noto, Michele Mussap, Mingbang Wang, Daniel N. Frank, Ellen Li, Wenhao Zhou, Vassilios Fanos, Valery N. Danilenko, Dennis P. Wall, Paúl Cárdenas, Manuel E. Baldeón, Sébastien Jacquemont, Omry Koren, Evan Elliott, Ramnik J. Xavier, Sarkis K. Mazmanian, Rob Knight, Jack A. Gilbert, Sharon M. Donovan, Trevor D. Lawley, Bob Carpenter, Richard Bonneau, Gaspar Taroncher-Oldenburg

**Affiliations:** 1 Center for Computational Biology, Flatiron Institute, Simons Foundation, New York, NY USA; 2 Biostatistics & Bioinformatics Branch, Eunice Kennedy Shriver National Institute of Child Health and Human Development, National Institutes of Health, Bethesda, MD USA; 3 Center for Genomics and Systems Biology, Department of Biology, New York University, New York, NY USA; 4 Precidiag, Inc., Watertown, MA USA; 5 Host-Microbiota Interactions Laboratory, Wellcome Sanger Institute, Hinxton, UK; 6 Bioinformatics and Systems Biology Program, University of California, San Diego, La Jolla, CA USA; 7 Department of Pediatrics, School of Medicine, University of California, San Diego, La Jolla, CA USA; 8 School of Life Sciences, Arizona State University, Tempe, AZ USA; 9 Biodesign Center for Fundamental and Applied Microbiomics, Arizona State University, Tempe, AZ USA; 10 Department of Electrical and Computer Engineering, University of California, San Diego, La Jolla, CA USA; 11 Department of Computer Science and Engineering, Jacobs School of Engineering, University of California, San Diego, La Jolla, CA USA; 12 Azrieli Faculty of Medicine, Bar Ilan University, Safed, Israel; 13 Laboratory for Research in Neuroimaging, Centre for Research in Neurosciences, Department of Clinical Neurosciences, Centre Hospitalier Universitaire Vaudois, University of Lausanne, Lausanne, Switzerland; 14 Division of Nutritional Sciences, University of Illinois, Urbana, IL USA; 15 Stark Neurosciences Research Institute, Indiana University School of Medicine, Indianapolis, IN USA; 16 Department of Anatomy, Cell Biology and Physiology, Indiana University School of Medicine, Indianapolis, IN USA; 17 Microbiology Institute and Health Science College, Universidad San Francisco de Quito, Quito, Ecuador; 18 Departments of Microbiology & Pharmaceutical Sciences, Oregon State University, Corvallis, OR USA; 19 Vavilov Institute of General Genetics Russian Academy of Sciences, Moscow, Russia; 20 Skolkovo Institute of Science and Technology, Skolkovo, Russia; 21 Department of Biomedical Sciences, School of Medicine, University of Cagliari, Cagliari, Italy; 22 Laboratory Medicine, Department of Surgical Sciences, School of Medicine, University of Cagliari, Cagliari, Italy; 23 Shanghai Key Laboratory of Birth Defects, Division of Neonatology, Children’s Hospital of Fudan University, National Center for Children’s Health, Shanghai, China; 24 Microbiome Therapy Center, South China Hospital, Health Science Center, Shenzhen University, Shenzhen, China; 25 Department of Medicine, University of Colorado Anschutz Medical Campus, Aurora, CO USA; 26 Department of Medicine, Division of Gastroenterology and Hepatology, Stony Brook University, Stony Brook, NY USA; 27 Neonatal Intensive Care Unit and Neonatal Pathology, Department of Surgical Sciences, School of Medicine, University of Cagliari, Cagliari, Italy; 28 Pediatrics (Systems Medicine), Biomedical Data Science, and Psychiatry and Behavioral Sciences, Stanford University, Stanford, CA USA; 29 Institute of Microbiology, COCIBA, Universidad San Francisco de Quito, Quito, Ecuador; 30 Facultad de Ciencias Médicas, de la Salud y la Vida, Universidad Internacional del Ecuador, Quito, Ecuador; 31 Sainte Justine Hospital Research Center, Montréal, QC Canada; 32 Department of Pediatrics, Université de Montréal, Montréal, QC Canada; 33 The Leslie and Susan Gonda Multidisciplinary Brain Research Center, Bar Ilan University, Ramat Gan, Israel; 34 Broad Institute of MIT and Harvard, Cambridge, MA USA; 35 Department of Molecular Biology, Massachusetts General Hospital, Boston, MA USA; 36 Center for the Study of Inflammatory Bowel Disease, Massachusetts General Hospital, Boston, MA USA; 37 Division of Biology & Biological Engineering, California Institute of Technology, Pasadena, CA USA; 38 Department of Bioengineering, University of California, San Diego, La Jolla, California USA; 39 Center for Microbiome Innovation, University of California, San Diego, La Jolla, California USA; 40 Scripps Institution of Oceanography, University of California, San Diego, La Jolla, CA USA; 41 Prescient Design, a Genentech Accelerator, New York, NY USA; 42 Gaspar Taroncher Consulting, Philadelphia, PA USA; 43 Simons Foundation Autism Research Initiative, Simons Foundation, New York, NY USA

**Keywords:** Microbiology, Data integration, Autism spectrum disorders, Genomics

## Abstract

Autism spectrum disorder (ASD) is a neurodevelopmental disorder characterized by heterogeneous cognitive, behavioral and communication impairments. Disruption of the gut–brain axis (GBA) has been implicated in ASD although with limited reproducibility across studies. In this study, we developed a Bayesian differential ranking algorithm to identify ASD-associated molecular and taxa profiles across 10 cross-sectional microbiome datasets and 15 other datasets, including dietary patterns, metabolomics, cytokine profiles and human brain gene expression profiles. We found a functional architecture along the GBA that correlates with heterogeneity of ASD phenotypes, and it is characterized by ASD-associated amino acid, carbohydrate and lipid profiles predominantly encoded by microbial species in the genera *Prevotella*, *Bifidobacterium*, *Desulfovibrio* and *Bacteroides* and correlates with brain gene expression changes, restrictive dietary patterns and pro-inflammatory cytokine profiles. The functional architecture revealed in age-matched and sex-matched cohorts is not present in sibling-matched cohorts. We also show a strong association between temporal changes in microbiome composition and ASD phenotypes. In summary, we propose a framework to leverage multi-omic datasets from well-defined cohorts and investigate how the GBA influences ASD.

## Abstract (web summary)

Autism spectrum disorder (ASD) is characterized by heterogeneous phenotypes. Disruption of the gut–brain axis (GBA) has been implicated in ASD although with limited reproducibility across studies. In this study, the authors propose a framework to leverage multi-omic datasets and investigate how the GBA influences ASD.

## Main

Autism spectrum disorder (ASD) encompasses a broad range of neurodevelopmental conditions defined by heterogeneous cognitive, behavioral and communication impairments that manifest early in childhood^1^. To date, over 100 genes have been identified as putatively associated with ASD, with some genotypes now having a standardized clinical diagnosis^2^. However, most of the genetic variants are still associated with heterogeneous phenotypes, making it difficult to identify molecular mechanisms that might be responsible for particular impairments^3^. Some studies have also looked at the presence of abnormalities in different brain regions in children with ASD^4^. However, whether such neuroanatomical features could mechanistically determine autism, and whether environmental factors could induce analogous ASD-like symptoms, remain unresolved^1^.

In addition to risk factors, one comorbidity that has been linked to ASD with high confidence is the occurrence of gastrointestinal (GI) symptoms, such as constipation, diarrhea or abdominal bloating, but causal insights remain elusive^5^. Mechanistically, much research has been focused on the interplay between the GI system and processes controlled by the neuroendocrine, neuroimmune and autonomous nervous systems, all of which converge around the GI tract and together modulate the gut–brain axis (GBA)^6^.

The GBA facilitates bidirectional communication between the gut and the brain, contributing to brain homeostasis and helping regulate cognitive and emotional functions^7,8^. Over the past decade, research on the factors modulating the GBA has revealed the central role played by the gut microbiome—the trillions of microbes that colonize the gut—in regulating neuroimmune networks, modifying neural networks and directly communicating with the brain^9^. Dysregulation of the gut microbiome and the ensuing disruption of the GBA are thought to contribute to the pathogenesis of neurodevelopmental disorders, including autism, but the underlying mechanisms and the extent to which the microbiome explains these dynamics are still unclear^10^.

Several dozen autism gut metagenomics studies have revealed many, albeit inconsistent, variations in microbial diversity in individuals with ASD compared to neurotypical individuals^10^. Similarly, metagenome-based functional reconstructions and metabolic analyses have also shown strong, albeit inconclusive, differences between individuals with ASD and neurotypical individuals^11^. Comparative analyses at other omic levels have further shown little agreement across studies^12^, raising the question of whether the results obtained so far reflect intrinsic biological differences among cohorts, insufficient statistical power or experimental biases that preclude meaningful comparisons^13^.

A wide range of factors could explain the disagreement across studies, including confounding variation due to batch effects, the application of inappropriate statistical methodologies and the vast phenotypic and genotypic heterogeneity of ASD. Batch effects can be caused by many factors, including mis-specified experimental designs, technical variability, geographical location and demographic composition, and several algorithms have been proposed to correct for them, but a lack of standardized statistical methods further complicates interpretation^14^. Microbiome datasets, like other omic datasets, are compositional, and failure to account for the compositional nature of sequencing counts can lead to high false-positive and false-negative rates when identifying differentially abundant microbes^15^. Microbiome analysis in ASD is further confounded by the phenotypic and genotypic heterogeneity of the disorder, which is known to be critical for stratifying ASD subtypes and constructing reliable diagnostics, but is typically not measured or controlled for^1^.

Understanding the functional architecture—the network of interactions among different omic levels that determines individual phenotypes—of complex neurodevelopmental disorders, such as autism, requires an accurate and comprehensive characterization of the different omic levels contributing to it^16^. Traditionally focused on the human genomic, metabolic and cellular components, mounting evidence of the role the GBA plays in phenotype determination raises the need for considering the metagenomic and metabolic contributions of the microbiome as potential key components of the functional architecture of autism^17^.

To identify autism-specific omic profiles while reducing cohort-specific confounding factors, we devised a Bayesian differential ranking algorithm to estimate a distribution of microbial differentials, or relative log fold changes^15^, across multiple potential ASD subtypes implicit in 25 omic datasets (Table 1). Ranking microbes by their log fold changes allows us to simultaneously (1) cancel out the compositional bias inherent in microbiome datasets and (2) minimize inflated false positives due to microbe-specific false discovery rate (FDR)-corrected statistical tests^15^. A key feature of our approach was to match individual study participants by sex and age within each study to adjust for confounders in childhood development. This setup also reduced confounding variation due to cohort-specific processing protocols, because within-study fold change calculations are insensitive to batch effects^18^ (Extended Data Fig. 2). The preponderance of autism among males is well documented, and several potentially sex-dependent mechanisms to explain this phenomenon have been proposed. Furthermore, the development of the microbiome during childhood is a hallmark of microbiome dynamics in the human gut. Our analysis reveals strong associations among omic levels along the GBA and in particular of the microbiome in the context of ASD. Ultimately, our analysis highlights the inherent limitations of cross-sectional studies for understanding the dynamics of the functional architecture of autism and provides a framework for future studies aimed at better defining the causal relationship between the microbiome and other omic levels and ASD.

**Table 1:** ASD omic datasets included in this study. All sequencing datasets were retrieved from the SRA

Organism	Body type	Data type	Number of studies	Number of subject pairs	References
Human	Postmortem brain tissue	RNAseq	4	49	Velmeshev et al. 2019 (ref. ^59^); Wright et al. 2017 (ref. ^60^); Herrero et al. 2020 (ref. ^61^); SRP072713 (ref. ^62^)
Human	Serum	Immune markers	1	22	Zurita et al. 2020 (ref. ^24^)
Human	Serum	Metabolome	2	50	Needham et al. 2021 (ref. ^49^); Kuwabara et al. 2013 (ref. ^48^)
Human	Urine	Metabolome	1	26	Noto et al. 2014 (ref. ^50^)
Human	NA	Dietary survey	1	26	Berding & Donovan 2019 (ref. ^25^)
Human	NA	Behavioral survey	1	28	Kang et al. 2019 (ref. ^52^)
Microbial	Fecal	Metabolome	2	43	Needham et al. 2021 (ref. ^49^); Kang et al. 2018 (ref. ^63^)
Microbial	Fecal	16S amplicon	10	346	Berding & Donovan 2019 (ref. ^25^); Zurita et al. 2020 (ref. ^24^); Dan et al. 2020 (ref. ^28^); Chen et al. 2020 (ref. ^22^); Fouquier et al. 2021 (ref. ^23^); Zou et al. 2020 (ref. ^27^); Kang et al. 2019 (ref. ^52^) and 2017 (ref. ^26^); Son et al. 2015 (ref. ^36^); David et al. 2021 (ref. ^37^); SRP299486 (ref. ^29^); Martin-Brevet et al.^38^
Microbial	Fecal	Shotgun metagenomics	3	83	Averina et al. 2020 (ref. ^34^); Wang et al. 2019 (ref. ^33^); Dan et al. 2020 (ref. ^28^)

## Results

**Fig. 1:**
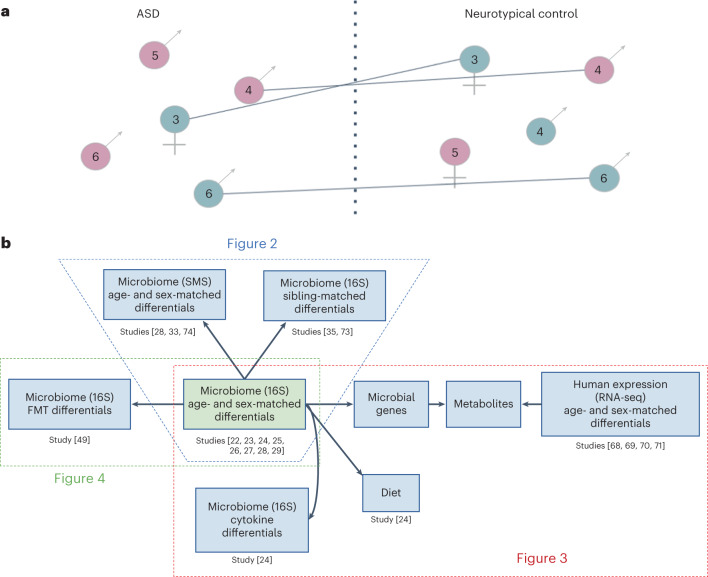
Diagram delineating the concept of age matching and sex matching. **a**, Children with ASD and neurotypical children of the same gender and similar age (±6 months) were matched within studies to reduce batch effects due to experimental and other cohort-specific differences. Matched pairs were then used to compute differentials (log fold ratios) of different omic features (microbes, metabolites, etc.). Downstream analyses across studies compared the within-study differentials determined for the different pairs of matched individuals (numbers inside circles denote age in years). **b**, The structure of our meta-analysis across multiple omic levels. For Fig. 2, 16S differentials computed from age-matched and sex-matched cohorts were cross-referenced against 16S differentials from sibling-matched cohorts as well as against SMS differentials from other age-matched and sex-matched cohorts. For Fig. 3, the 16S differentials from the age-matched and sex-matched cohorts were cross-referenced against cytokine differentials and RNA-seq differentials using KEGG pathways as a reference. Figure 3 also includes a microbe–diet co-occurrence analysis. For Fig. 4, the 16S differentials from the age-matched and sex-matched cohorts were cross-referenced against 16S differentials computed from the Kang et al. FMT trial^52^.

**Extended Data Fig. 1:**
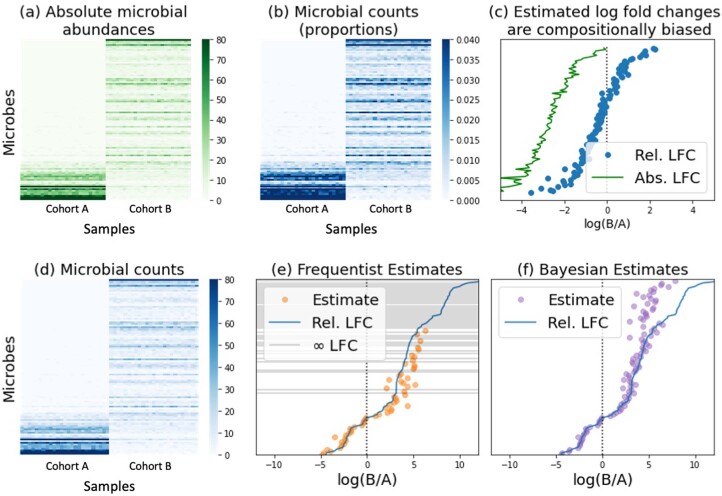
Study approach. Metagenomic sequence data present unique quantification challenges due to a lack of total microbial load measurements, which precludes the determination of absolute microbe abundances, and to limitations brought about by sampling and sequencing depth limitations, which result in an incomplete representation of the metagenome. We devised a Bayesian differential ranking algorithm to address both these challenges, the compositional challenge and the zero-inflation challenge. **The compositional challenge**: Most sequencing count datasets lack absolute abundance information in the form of cells, colony forming units, or transcripts per volume. This limitation preempts the reliable estimation of log fold changes (LFCs) and is a defining characteristic of compositional data that can lead to excessive false positives or false negatives depending on the magnitude of the change in absolute abundances^15^. As illustrated in panels a) through c), microbial counts (a) are typically converted into proportional abundances (b) that are then used to compute log-fold ratios. Fold change calculations adopt the general formula $\frac{B}{A}=\frac{{N}_{B}{p}_{B}}{{N}_{A}{p}_{A}}=\frac{{p}_{B}}{{p}_{A}}\times \frac{{N}_{B}}{{N}_{A}}$, where *A* and *B* represent the two samples being compared, *p*_*A*_ and *p*_*B*_ represent the microbial proportions in *A* and *B*, and *N*_*A*_ and *N*_*B*_ represent the total number of microbes in *A* and *B*, also known as the ground truth. A key limitation of sequencing count data is their lack of proportionality to the corresponding absolute abundances in the original samples due to sequencing depth constraints. Our inability to observe *N*_*A*_ and *N*_*B*_ introduces a bias that ultimately prevents us from performing false discovery rate (FDR) correction to identify differentially abundant microbes. This bias depends on the change in microbial population size, with large population shifts leading to increased false positive and false negative rates, and an overall skewed representation of the ground truth (c). **The zero-inflation challenge**: Sampling errors and shallow sequencing lead to disproportionately high numbers of zero counts, especially for microbes present in low abundances (d). Multinomial, Poisson and Negative Binomial distributions have been used to explicitly handle zero counts^21^. However, estimating log-fold differentials remains problematic when microbes are not observed in any of the samples in one group since log 0 is − *∞* and thus the true log-fold change of a zero-count microbe can not be determined (e). Bayesian inference avoids this problem by introducing a prior that prevents nonsensical log-fold change estimates (f). Specifically, this introduces a rounded-zero assumption whereby all microbes have a non-zero chance of being observed. Panel h highlights what these log-fold changes would look like using a Dirichlet prior, where every microbe has the same probability of being observed before collecting data.

The structure of our analysis consisted of a multi-cohort and multi-omic meta-analysis framework that allowed us to combine independent and dependent omic datasets in one integrated analysis^19^. To minimize issues of compositionality and sequencing depth^20^, we modeled sequencing count data using a negative binomial distribution^21^ (Extended Data Fig. 1, ‘Study approach’). Our differential ranking approach incorporated a case–control matching component that individually paired children with ASD with age-matched and sex-matched neurotypical control children within each study cohort to adjust for confounding variation and batch effects (Supplementary Information). Finally, we cross-referenced the microbial differential rankings estimated from 16S rRNA gene (16S) amplicon data obtained from seven age-matched and sex-matched cohorts against 15 other omic datasets to contextualize the potential functional roles that these microbes could play in autism (Fig. 1).

### Age matching and sex matching enhance ASD data analysis

To establish the validity and robustness of our age-matched and sex-matched Bayesian differential ranking approach, we performed a series of benchmarking exercises and sensitivity tests.

**Extended Data Fig. 2:**
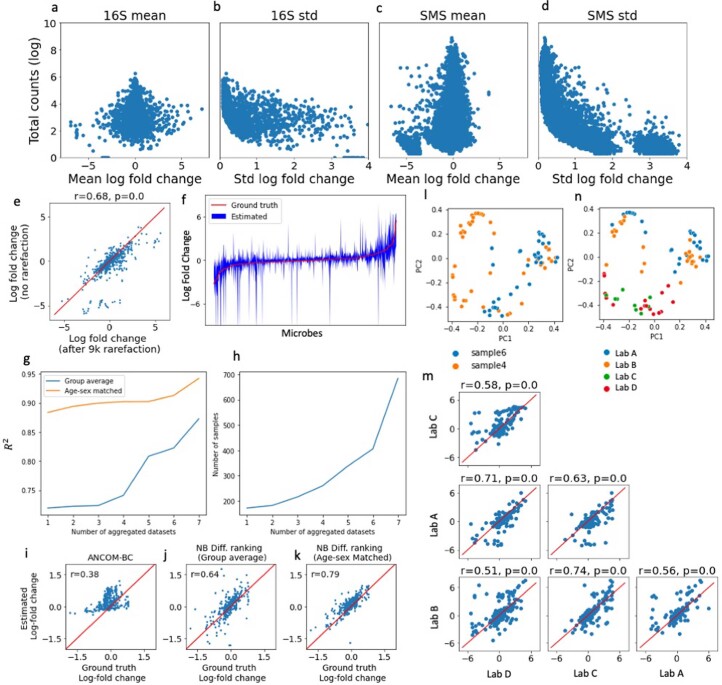
Benchmarks. (a-d) Mean and standard deviations of the per-microbe log-fold changes compared to the total sequencing depth (log10 scale) for each microbe. e) Rarefaction benchmark, showcasing how differential abundance analysis is insensitive to rarefaction. (f) Differential abundance estimation derived from a data-driven simulated 16S dataset. (g) Comparison of age- and sex-matching approach compared to standard group averaging with respect to dataset size across 7 of the 11 16S studies (excluding Kang et al^52^, David et al^37^ and Son et al^36^ The x-axis represents the number of aggregated datasets, the y-axis on the left panel is the average *R*^2^ metric to measure the model error. (h) Number of samples analyzed on the y-axis, and the x-axis on the right panel is the number of aggregated dataset. (i-k) Simulated datasets with a sequencing depth differential between matched cases and controls, where matched controls always have a larger sequencing depth than their case counterparts. This benchmark investigates how well ANCOM-BC, group averaged differential ranking and age-sex matched differential ranking can recover the ground truth log-fold changes. The group averaged and age-sex matched differential ranking both use the Negative Binomial (NB) distribution to model sequencing count data.(l-m) Simulated datasets comparing household matching to age-sex matching. (l-n) Bray-Curtis PCoA of 2 samples replicated across 4 processing labs in the MBQC^31^. (m) Pairwise comparsions of log-fold change between 2 samples across all 4 labs using group-averaged differential abundance analysis.

We started by investigating the means and the standard deviations for the 16S and shotgun metagenomics sequencing (SMS) differentials from the age-matched and sex-matched cohorts compared to the total sequencing depth for each microbe (Extended Data Fig. 2a–d). In both analyses, we observed that the models could use sequencing depth to calibrate the uncertainty estimates, giving larger standard deviations for rare taxa with fewer observed reads (Extended Data Fig. 2b,d). Furthermore, rare taxa (with fewer than 100 reads total) were not among the most differentially abundant ASD-associated taxa (Extended Data Fig. 2a,c).

Next, we performed a rarefaction benchmark to test whether the high frequency of rare taxa would influence the results of our log fold change calculations. A comparison of differential abundance estimates between rarefied (9,000 threshold) and non-rarefied data from our 16S cross-sectional datasets showed that rarefaction did not substantially affect our results (Extended Data Fig. 2e). We also conducted a data-driven simulation with varying differential sequencing depths between cases and controls and showed that, despite the sequencing depth confounder, our differential abundance method could accurately recover the ground truth log fold changes (Extended Data Fig. 2f). We then compared performance of our age-matched and sex-matched differential ranking analysis to the standard group-averaged differential ranking analysis across seven out of the 11 16S studies^22–29^ (Extended Data Fig. 2g). A side-by-side comparison with a commonly used differential abundance approach, ANCOM-BC (ref. ^30^), was then conducted to highlight the differences between our methodology and one of the state-of-the-art differential abundance methods (Extended Data Fig. 2i–k).

We benchmarked the overall batch-effect-reducing power of performing within-study differential analyses with our sex-matched and age-matched Bayesian differential ranking approach. We used the MicroBiome Quality Control (MBQC) study (Sinha et al. 2017 (ref. ^31^)) to evaluate the extent to which within-study differential analysis removed experimental and other study-related confounders, allowing for meaningful comparisons across independent studies. Focusing on the microbial abundance datasets (16S microbial counts) generated by four independent laboratories (Lab A, Lab B, Lab C and Lab D) processing two identical MBQC microbiome samples (samples ‘4’ and ‘6’), we calculated the differentials between microbial counts for these samples for each laboratory. An initial assessment of overall variability between the two samples (principal coordinate analysis (PCoA) plot with Bray–Curtis dissimilarity) (Extended Data Fig. 2l) showed a reasonable separation between both samples just based on microbial counts, but a visualization of study membership revealed that a significant degree of variability was associated with the laboratory that generated the dataset (Extended Data Fig. 2n). Given that the metagenomic samples by each laboratory were identical, the high level of variability observed among datasets could be ascribed only to experimental and laboratory-specific batch effects. Consistent with the theoretical findings of McLaren et al.^18^, our differential analysis showed a high degree of correlation among within-study differentials, clearly supporting the use of scale-equivariant log fold change calculations within studies as a way to provide a high-confidence readout of ground truth differentials and to enable cross-comparisons of independent cohort studies (Extended Data Fig. 2m).

To determine if our analyses can generalize between 16S and SMS datasets, we focused on the Dan et al.^28^ cohort that paired 16S and SMS samples. Abundances obtained after mapping reads to the Greengenes2 database^32^ highlight strong agreement between 16S and SMS datasets on the genus level (*r* = 0.63, *P* < 1 × 10^−100^). Furthermore, the log fold changes between 16S and SMS obtained from our age-matching and sex-matching approach also show strong agreement on the genus level (*r* = 0.47, *P* = 1 × 10^−7^).

### Differential ranking analysis reveals strong ASD–microbiome links

**Fig. 2:**
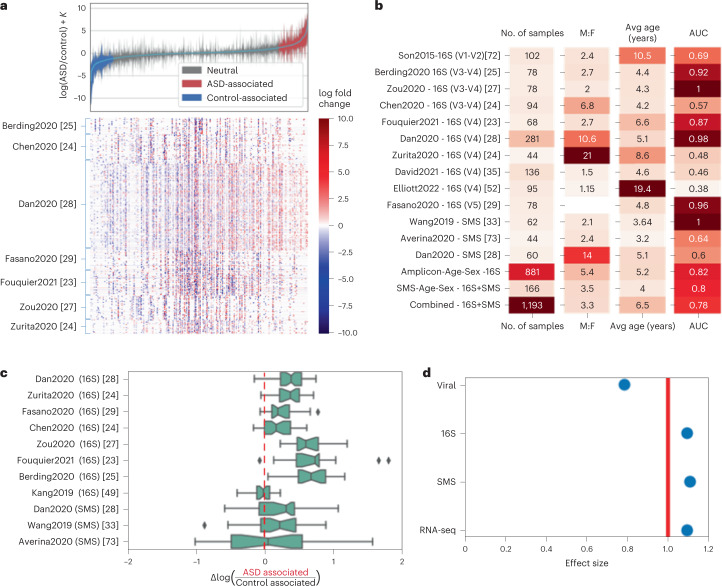
Differential ranking analysis across omics levels. **a**, Global microbial 16S log fold changes between age-matched and sex-matched ASD and control individuals. Error bars represent the 95% credible intervals. Heat map showing all center log ratio (CLR) transformed microbial differentials for each age-matched and sex-matched ASD–control pair across all cohorts. Microbes are binned into ASD-associated, Neutral and Control-associated groups using an age-matched and sex-matched classifier (Methods). *K* is an unknown bias due to the shift in the microbial load between the ASD and neurotypical control population. **b**, Sample size, male:female (M:F) ratio and average ages across all 16S and shotgun metagenomics datasets analyzed in this study and held-out gradient boosting ASD prediction performance measured by AUROC. V3–V4, V4 and V4–V5 refer to the variable region of the bacterial ribosomal RNA analyzed. **c**, Log ratios of microbes that are classified to be ASD associated and control associated were computed for each sample. The *x* axis represents the case–control differences of these log ratios, where values greater than 0 indicate that there is a separation between children with ASD and neurotypical children. The box plots show the median (line), 25–75% range (box) and 5–95% range (whiskers). **d**, Effect sizes of different omics levels: viral, 16S, SMS and RNA-seq.

**Extended Data Fig. 3:**
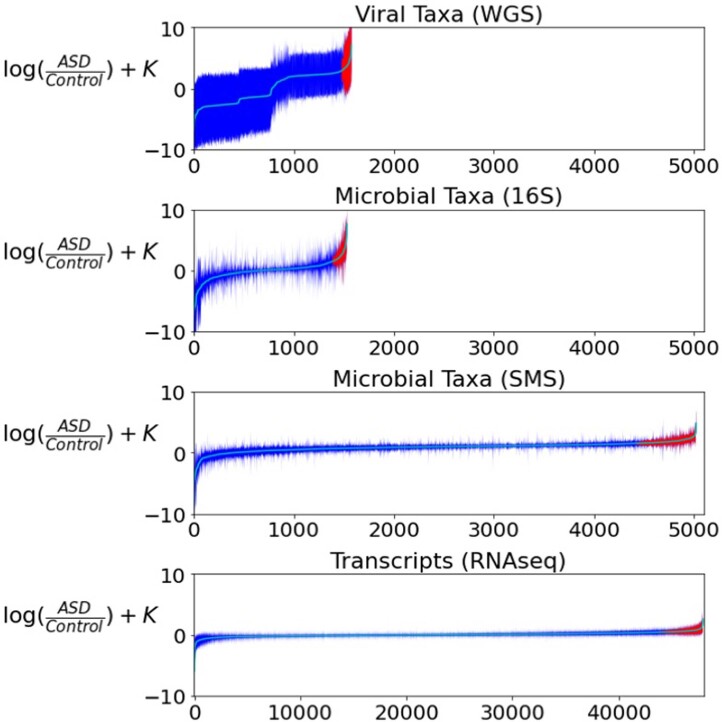
Differential ranking trends observed for the virome, 16S, SMS, and RNAseq datasets analyzed in this study. The top 10% most differentially abundant features are highlighted in red. The x axis for the virome, 16S and SMS datasets is equivalent to showcase the differences in feature counts; the x axes for the RNAseq dataset is larger by a factor of 10, illustrating the stark difference in number of features of this dataset compared to the other three.

A global age-matched and sex-matched differential ranking analysis of the seven 16S datasets selected for this study revealed a clear partitioning of microbial differences with respect to ASD and cohort membership (Fig. 2a). The distribution of the overall case–control differences showed a strong ASD-specific signal driven by 591 microbes more commonly found in children with ASD and 169 microbes more commonly found in their control counterparts (Supplementary Table 4). The variability observed is most likely due to confounding factors such as cohort demographics and geographic location, with the seven cohorts originating from Asia, Europe, South America and North America. Analogous differential ranking trends could be observed for the virome, SMS and RNA sequencing (RNA-seq) datasets (Extended Data Fig. 3). To determine whether these highly significant microbiome signals (*P* < 0.0025) could be used to distinguish children with ASD from their age-matched and sex-matched control counterparts, we trained random forest classifiers on train/validation/test splits of data derived from 16S-targeted sequencing and SMS–whole-genome sequencing of microbial communities. We fitted gradient boosting classifiers on combined microbiome datasets as well as on individual datasets and measured their performance with area under the receiver operating characteristic (AUROC) curve. Of the nine age-matched and sex-matched cohorts^22–25,27–29,33,34^, six of the studies had an AUC > 0.87, highlighting the strong microbial differences between children with ASD and neurotypical children within age-matched and sex-matched cohorts (Fig. 2b). The classification performance decreased when we trained one classifier across all 1,193 samples across all of the cohorts but is still predictive of ASD (AUC = 0.78). This is consistent with previous observations in other disease meta-analyses^35^, where within-study classification performance is greater than across-study classification performance. We suspect that widespread microbial heterogeneity across diverse human populations could play a role in impeding classification performance.

In contrast to the age-matched and sex-matched cohorts, the AUC dropped substantially in the sibling-matched cohorts (Son et al.^36^ AUC = 0.69; David et al.^37^ AUC = 0.46; Elliot et al.^38^ AUC = 0.38). Similarly PERMANOVA detected ASD-specific microbiome differences only in the age-matched and sex-matched cohort (*P* = 0.002), whereas no such signal was found in the sibling-matched cohort (*P* = 0.535). In both cohorts, age and sex were significant confounders (*P* < 0.002), but only in the age-matched and sex-matched cohort could the age and sex differences between case–control pairs be minimized (Extended Data Fig. 6a–d), where more than two times more case–control pairs are the within 1-year age difference and the same gender compared to the sibling-matched cohort (Extended Data Fig. 6e,f). Although household has been observed to be a confounder in the sibling-matched cohort (*P* < 0.001), we did see strong classifier generalization in the age-matched and sex-matched cohorts, where none of the children live in the same home. However, it is possible that unmeasured confounders, such as household diet or socioeconomic status, could artificially boost classification performance. To investigate the potential age and sex confounders in the sibling studies, we performed a data-driven simulation with a known ground truth to determine how large age differences (± >2 years) would bias modeling outcomes compared to optimized age matchings (± ≤0.5 years) using a sibling-like age distribution (obtained from David et al.^37^) and our overall sex-matched and age-matched distribution, respectively (Extended Data Fig. 6c). The analysis showed that, in case–controls with a sufficiently large age confounder, methods using age and sex matching or sibling matching cannot exactly recover the ground truth log fold changes. However, for sibling-like age distribution, the estimated log fold changes exhibited a large bias (mean squared error = 589.3) that was reduced by an order of magnitude in the sex-matched and age-matched group (mean squared error = 57.8) (Extended Data Fig. 6g,h).

Age-matched and sex-matched differential analysis outperformed standard group averaging with respect to R^2^, and its overall performance strictly improved as more studies were added (Extended Data Fig. 2g,h). This performance boost reflected a reduction in model uncertainty with larger cohorts that was indicative of overlapping differentially abundant taxa across studies and of reduced confounding variation. To aid in the interpretation of the classification results, we constructed log ratios of taxa derived from the age-matched and sex-matched differential abundance analysis that strongly separated children with ASD from the neurotypical controls within each study. From these individual analyses, we assembled a single microbial log ratio that highlighted a strong consistent enrichment of taxa in children with ASD relative to their control counterparts with log ratios greater 0 across 88% of pairs (Fig. 2c). This pattern was consistent across all age-matched and sex-matched cohorts, including two held-out shotgun metagenomics datasets: Wang et al.^33^ (log ratios > 0 in 70% of pairs) and Dan et al.^28^ (log-ratios > 0 in 73% of pairs).

### ASD-specific patterns are present at several omic levels

**Extended Data Fig. 4:**
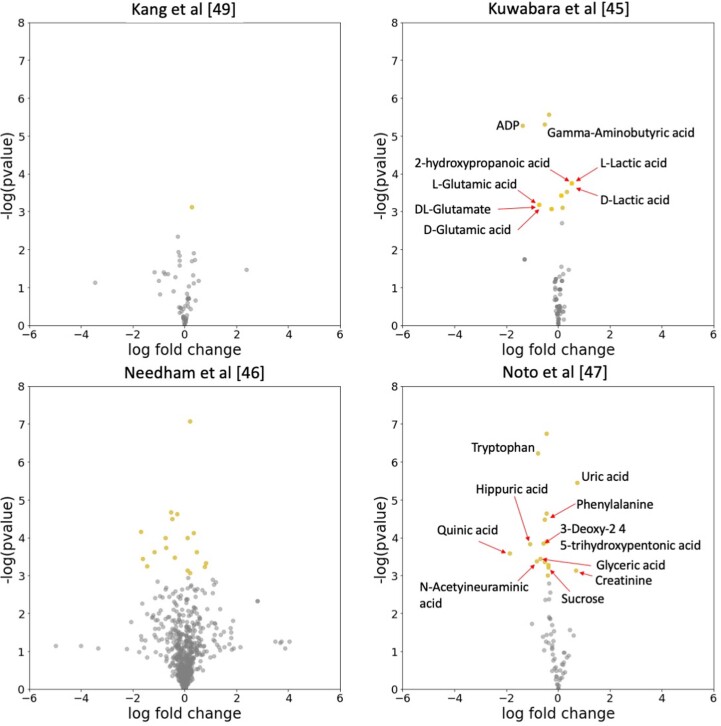
Metabolomics differential ranking analysis across four studies. Paired *t*-tests were performed to identify differentially abundant metabolites. The metabolites shown in Needham et al consist of both fecal and serum metabolites. None of the metabolites had significant log-fold changes after applying FDR correction.

Differential ranking analysis of three omic levels—microbiome (16S and SMS) and human transcriptome (RNA-seq)—revealed strong and highly significant differences between children with ASD and their age-matched and sex-matched neurotypical counterparts (*P* < 0.0025) (Fig. 2d and Supplementary Tables 5 and 6). Two additional omic levels—the metabolome and the virome—did not show significant signals (Extended Data Fig. 4 and Supplementary Table 7).

### Host cytokines correlate with microbial abundances

**Fig. 3:**
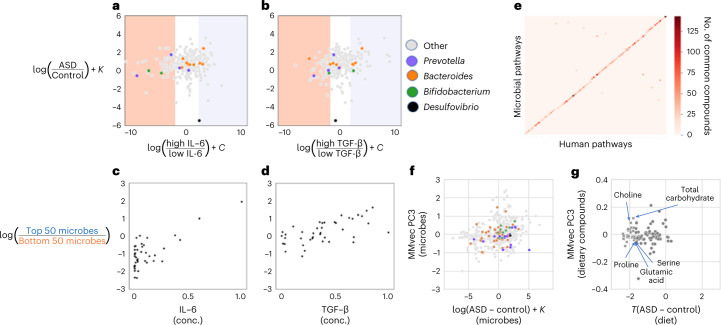
Characterizing the associations among differentially abundant microbes in ASD and cytokines, gene expression in the brain and dietary patterns. **a**,**b**, Comparison of microbial differentials obtained from age matching and sex matching and cytokine analysis. **c**,**d**, Microbial log ratios constructed from the 50 top and bottom most differentially abundant microbes corresponding to each cytokine. *K* and *C* represent unknown biases due to the shift in the microbial load between the ASD and neurotypical control population. **e**, Heat map showing the overlap of molecules between ASD-enriched pathways in the microbiome and in the brain. The microbial and human pathways are both sorted alpha-numerically; the dense diagonal is largely indicative of common pathways between microbial and human genomes. **f**,**g**, PC3 from microbe–diet co-occurrence analysis is contrasted against microbial log fold changes and dietary differences from Berding et al.^25^ Dietary compounds that are depleted (*P* < 0.1) in children with ASD are highlighted as ‘x’ markers. *T*(ASD-Control) represents the *t*-statistic that measures the differences between ASD and neurotypical dietary intake. conc., concentration.

Immune dysregulation, ranging from circulating ‘anti-brain’ antibodies and perturbed cytokine profiles to simply having a family history of immune disorders, has been repeatedly associated with ASD^39^. Recently, for example, Zurita et al.^24^ showed that concentrations of the inflammatory cytokine transforming growth factor beta (TGF-β) are significantly elevated in children with ASD. We re-analyzed this dataset, after age matching and sex matching, and observed that 16S microbial differentials estimated from Zurita et al.^24^ were associated with TGF-β and were positively correlated with the global microbial log fold changes between ASD and control pairs (TGF-β: *r* = 0.237, *P* = 2.84 × 10^−5^) (Supplementary Tables 1 and 9). In contrast, the global microbial log fold changes had little correlation with interleukin (IL)-6 concentrations (*r* = 0.07, *P* = 0.17). However, when we calculated the log ratios of the most differentiating microbial taxa, they were highly correlated with both TGF-β and IL-6 concentrations (TGF-β: *r* = 0.61, *P* = 1.84 × 10^−5^; IL-6: *r* = 0.73, *P* = 5.74 × 10^−8^) (Fig. 3a–d). This highlights how IL-6 changes are linked to only a handful of taxa, whereas TGF-β is linked to a much larger number of taxa.

*Prevotella*, *Bacteroides* and *Bifidobacterium* were predominantly associated with the cytokine differentials. Partial mechanistic insights on some of these cytokine–microbe associations were previously published. *Bacteroides thetaiotaomicron* was the second most highly elevated microbe when TGF-β was depleted and has been suggested to play a role modulating maternal immune activation-dependent metabolites that are linked to behavorial symptoms^40^. *Bifidobacterium callitrichidarum* was the sixth most enriched taxon when IL-6 was in lower concentration. Other *Bifidobacteria* species, such as *Bifidobacterium longum*, have been observed to downregulate IL-6 in fetal human enterocytes in vitro^41^. *Prevotella copri* was the second most enriched taxon when IL-6 was in lower concentration and the sixth most enriched taxon when TGF-β was in lower concentration. This was consistent with Tett et al.^42^, where *P. copri* associations with different cytokines were observed in multiple disease contexts. Similarly, *P. copri* and *Bacteroides fragilis* both co-occurred with phages enriched in children with ASD or in neurotypical children (Extended Data Fig. 7 and Supplementary Table 10), but, whereas microbes were previously reported to mediate viral infections^43^, the mechanistic underpinnings of these interactions with the host’s immunity remain poorly understood^44^.

### Microbiome metabolism mirrors human brain metabolism in ASD

To determine potential crosstalk between microbiome physiology and the human brain, we compared the metabolic capacities encoded by the microbial metagenome—combining the individual metabolic capacities of thousands of different microbes—and the differentially expressed human genome in the brain, two omic levels representing entirely different biological contexts. We identified 138 microbial and 1,772 human metabolic encoding genes, inferred from SMS and RNA-seq, respectively, that were linked to ASD phenotype. Ninety-five human metabolic pathways differentially expressed in the brain tissues of individuals with ASD had analogous microbial pathways differentially abundant in the microbiome of children with ASD, suggesting a potential coordination of metabolic pathways across omic levels in ASD (Fig. 3e). Pathways related to amino acid metabolism, carbohydrate metabolism and lipid metabolism were disproportionately represented among the overlapping pathways (Extended Data Fig. 9 and Supplementary Table 14). Cross-comparison of the ASD-associated microbial enzyme-encoding genes with the gut–brain modules (GBMs), previously defined as part of the GBM framework, also revealed an approximately 48.5% overlap (101/208), further supporting the notion of potential metabolic crosstalk across omic levels^45^ (Supplementary Table 13).

### Microbiome metabolic capacity mirrors diet patterns in ASD

Autistic traits in early childhood have been shown to correlate with poor diet quality later in life; however, little is known about how diet quality is directly linked to autistic traits^46^. Here, we re-analyzed the paired microbiome and dietary survey data from Berding et al.^25^. A microbiome–diet co-occurrence analysis revealed startlingly similar amino acid, carbohydrate and lipid metabolism association patterns to those observed in the microbiome–brain metabolic capacity analysis (Supplementary Tables 2 and 15) (*Q*^2^ = 0.43). From the microbe–diet co-occurrence analysis, only principal component (PC) 3, which explains 3% of the microbe–diet variance, could differentiate between ASD and neurotypical diets (*r* = 0.26, *P* = 0.004) and was strongly correlated with the microbial log fold changes between ASD and age-matched and sex-matched controls (*r* = 0.22, *P* = 4.3 × 10^−9^). Autistic children were less likely to consume foods high in glutamic acid, serine, choline, phenylalanine, leucine, tyrosine, valine and histidine, all compounds involved in neurotransmitter biosynthesis^47^. Interestingly, multiple *Bacteroides* taxa and *P. copri* taxa were among the top 20 taxa along MMvec PC3, highlighting how these taxa could be involved in metabolizing amino acid dietary compounds (Fig. 3f and Extended Data Fig. 8). Even though the metabolomic analysis did not yield statistically significant signals after FDR correction, the metabolites that showed the strongest signal included glutamate and phenylalanine, consistent with the microbiome–diet analysis^48–50^. Disruptions in the biosynthesis of these neurotransmitter molecules have been implicated in a wide variety of psychiatric disorders, and a recent blood metabolomics study showed the potential of using branched-chain amino acids to define autism subtypes^51^. Due to the incompatibility among the molecular features across datasets, it was not possible to combine any of the metabolomics datasets to boost the statistical power, which remains a major limitation of metabolomics technologies at present (Methods).

### ASD microbiomes mirror behavior improvement after fecal matter transplant

Although the preceding cross-sectional analyses showed significant associations among several omic levels (virome, microbiome and immunome) or diet and ASD, insights into causality are still limited. By contrast, longitudinal intervention studies provide an opportunity to obtain stronger insights into causality. To test this, we re-analyzed data from a 2-year, open-label fecal matter transplant (FMT) study with 18 children with ASD^52^. In this study, the children were subjected to a 2-week antibiotic treatment and a bowel cleanse, followed by 2 d of high-dose FMT treatment and 8 weeks of daily maintenance FMT doses. Based on one of the most common evaluation scales for ASD, the Childhood Autism Rating Scale (CARS), significant improvements were achieved after the 10-week course of treatment. Two months later, the initial gains were largely maintained, and a 2-year follow-up showed signs of further improvement in most of the patients. The results are consistent with a potential role of the microbiome in improving autism symptoms, but how the underlying changes in microbiome composition related to those seen in other studies remains unknown.

**Fig. 4:**
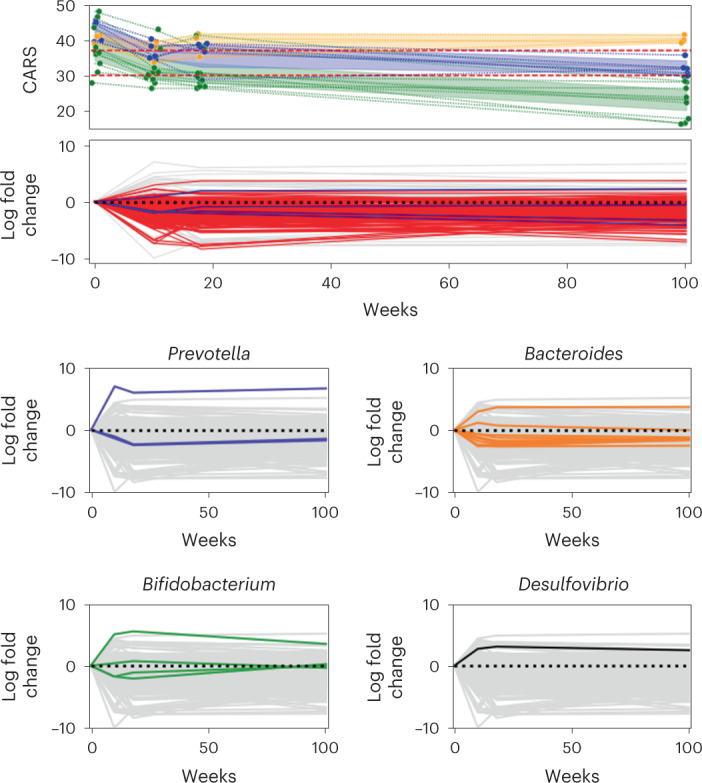
FMTs have long-lasting effects on autism gut microbiomes. **a**, The improvement of CARS for each child with ASD over time. The children were split into three groups—non-ASD, mild/moderate and severe—based on whether their CARS score fell below 30, was between 30 and 37 or was higher than 37. **b**, Microbial log fold changes over time: the time series was generated by calculating log fold changes between timepoints for each microbe. ASD-specific microbes highlighted in red were determined in the cross-sectional study. **c**–**f**, Microbial log fold changes are re-colored with genera highlighted in cytokine comparisons.

In the present study, we re-analyzed the original raw data in the context of the ASD profiles revealed by our cross-sectional differential ranking analysis (Supplementary Tables 3 and 16). All microbes associated with ASD in the 18 children before the FMT treatment had been identified as ASD-associated microbes in our age-matched and sex-matched cross-sectional analysis. After 2 years, 91% of these microbes that had low uncertainty (posterior standard deviation < 3) exhibited a mean decrease in abundance, and this decrease was significant (95% log fold change quantile < 0) in 57% of the microbes (Fig. 4). Consistent with the original analysis by Kang et al., we detected an increase in *Prevotella sp*. over the 2-year span of the study. In addition, we also determined an increase in *Desulfovibrio piger* and no significant changes in *Bifidobacteria*, counter to the original analysis by Kang et al.^52^ Interestingly, 305 taxa remained stable throughout the duration of the study. Of these, 13 taxa belonged to the *Prevotella*, *Bifidobacterium*, *Bacteroides* and *Desulfovibrio* lineages, pointing to a potentially wide functional diversity within these genera not noted in the original study. Some of these taxa, including *B. fragilis*, *B. thetaiotaomicron*, *B. longum* and *P. copri*^42^, were previously associated with beneficial immunomodulatory properties. Also worth pointing out are multiple butyrate producers in the *Butyricimonas* and *Anaerobutyricum* genera that we detected as being stable throughout the 2 years of the study, indicating a potential role in contributing to GBA homeostasis^53^.

## Discussion

The functional architecture of ASD, and in particular the potential role that the microbiome plays in modulating the GBA in the context of autism, remain poorly understood due to disagreements among existing microbiome and other omic studies. However, in contrast to recently reported findings^54^, we observed a clear separation between children with ASD and unrelated age-matched and sex-matched neurotypical controls, and this signal was validated using three distinct methodologies—namely PERMANOVA, classification and differential abundance—across multiple cohorts. Unlike the age-matched and sex-matched analysis, no ASD–microbiome signal was detected in the sibling-matched cohorts, including in the Elliott et al.^38^ dataset that consists of individuals with chromosome 16p11.2 deletion, a known risk factor for ASD. One possibility is due to age and sex confounding in the sibling cohorts, because age remains a major confounding factor in early childhood microbiome development^55^. However, we cannot rule out the possibility that our classifiers are identifying differences between households rather than individuals with ASD in the age-matched and sex-matched cohorts. Previous efforts identified household-specific effects on the human microbiome^56^, and other studies raised issues with sibling controls in these studies because siblings often exhibit a higher risk of developing ASD compared to the general population^57^. However, the fact that we see a clear ASD–microbiome signal that generalizes across households within cohorts highlights the need to control these confounding factors to understand the functional role that these gut microbiota could play. Thus, a follow-up study investigating gut microbiome and genetic variation between households with and without children with ASD is needed.

Parallel analyses at the immunome, human transcriptome and dietome levels revealed strong associations among omic levels. The virome and the direct metabolome signals, although present, were markedly weaker than the other omic signals. The inferred ASD-specific metabolic profiles from the microbiome and the human transcriptome, on the other hand, showed a high and significant degree of overlap in microbial and human pathways expressed in the gut and in the brain, respectively. The metabolic connection implied by this overlap, which included differentially enriched carbohydrate and amino acid metabolic pathways in ASD, is a remarkable observation given the fundamental difference between the gut and brain physiologies, which would a priori suggest a reduced overlap in metabolic capacities. The microbiome–diet co-occurrence analysis also highlighted a reduced intake of amino acids and carbohydrates linked to specific microbiome profiles in children with ASD. These metabolic and dietary imbalances, particularly regarding glutamate levels, were further apparent, albeit weakly, in the serum, fecal and urine metabolomes that we analyzed. This multi-scale overlap that we observed along the GBA points to the existence of a functional architecture of ASD driven by the metabolic potential at the genomic and metagenomic levels.

In light of the heterogeneity across studies, our analysis identified several microorganisms consistently detected across omic levels that point to potentially interesting functional connections. The diet co-occurrence analysis also showed a strong association between *P. copri* and carbohydrate depletion in ASD, in addition to upregulation of IL-6. *Bacteroides* genera are observed to play a key role in ASD diet differentiation, with *B. thetaiotaomicron* associated with the depletion of TGF-β. Multiple other microbes, including *P. copri* and several *Bacteroides*, stood out in the immune and viral analyses. In the FMT study, we observed a stable core microbiome made up of *Bacteroides*, *Prevotella*, *Bifidobacteria* and *Desulfovibrio* in addition to multiple butyrate producers. The presence of this core microbiome in combination with the depletion of most ASD-associated taxa further suggests a causal role for these microorganisms in shaping autism symptoms.

Despite our inability to determine actual metabolomic profiles at this point (Methods), our metabolite analysis based on microbiome-derived and brain-derived metabolite inferences as well as the diet-derived metabolite data reveals a picture of a unifying and distinct ASD functional architecture. With the brain, the immunome and diet as major effectors, the multi-factorial complexity of ASD is reduced to a multi-scale set of interactions centered around human and bacterial metabolism that, in turn, determines phenotypic, genomic and metagenomic attributes via multiple feedback loops. Although we did not observe an effect on genotype to the microbiome, previous studies identified genes that are high risk for ASD^2^. The pivotal role of the immune system in mediating the communication between the gut microbiome and the human brain as well as other peripheral systems is also firmly established. Furthermore, the central role of the microbiome in mediating diet-derived nutrient mobilization has been extensively documented, and several hardwired feedback loops among these effectors, such as the hypothalamus-mediated regulation of appetite and diet, have also been described^6^.

Our understanding of how the gut microbiome is connected to dietary preferences, host immunity and GI and ASD behavioral symptoms is limited in cross-sectional studies and, thus, restricts our ability to perform causal inference. We envision that obtaining causal insights into the functional architecture of autism will require a multi-arm approach, from culturing key microbes and probing their metabolic capacity, to performing experimental interventions with model organisms and conducting longitudinal observational studies, with multi-omics data collection and extensive phenotypic profiling to observe the effects of natural interventions. Building realistic causal models of autism needs to take into account the multi-factorial complexity underlying different ASD subtypes, which will require a concerted effort to simultaneously analyze several omic levels and at clinically relevant timescales. For instance, understanding the engraftment dynamics of FMT and its functional implications on the recipients’ gut microbiomes requires frequent initial sampling of the microbiome, immunome and metabolome, but tracing any behavioral changes over time requires less frequent sampling over periods of up to several years, in combination with reliable behavioral, medical and dietary surveys^58^. Collecting and integrating such multi-scale omic datasets presents unique logistical and analytical challenges.

Managing data acquisition and access will require coordinating multiple sites and potentially centralizing some aspects of sample processing. Recent initiatives, such as The Environmental Determinants of Diabetes in the Young (TEDDY) study, an international long-term, multi-center initiative to link specific environmental triggers to particular type 1 diabetes-associated genotypes, provide a blueprint for similar approaches in ASD. A key component of such an initiative would be the establishment of standardized sampling and processing protocols that would minimize technical confounders, one of the top confounders at most omic levels. Moreover, although extensive efforts are underway to calibrate microbiome datasets, other omic levels, such as the metabolome, present even more fundamental technical issues that make it imperative to develop concerted strategies to be able to include them in an integrated analysis.

In addition to the considerable variations in statistical properties across datasets, interactions among omic levels are mostly underdetermined, making the construction of informative models a major challenge. Determining the necessary biologically relevant assumptions is a non-trivial process and can inadvertently lead to model mis-specifications, resulting in misleading conclusions. This was the likely consequence from Yap et al.^54^, where the proposed model that tested for a causal relationship among diet, microbes and the ASD phenotype implicitly assumed that there was no relationship between diet and gut microbiome, prematurely rejecting the potential role between gut microbiota and ASD. Addressing these types of model mis-specication issues will be critical to inferring causal mechanisms from population-scale studies. In addition, and given the vast heterogeneity of ASD, designing cohort studies that minimize confounding factor effects will be key to furthering understanding of autism. For example, although our analysis could not identify ASD subtypes, we determined stronger associations among gut microbes, host immunity, brain expression and dietary patterns than previously reported, highlighting the potential for boosting the statistical power and biological insight with comprehensive omic analyses.

We conclude that multi-omic longitudinal intervention studies on appropriately stratified cohorts, combined with comprehensive patient metadata, would provide the necessary entry points for advancing mechanistic studies along the GBA in ASD. The experimental framework that we propose for inferring causal mechanisms from population-scale studies will require the development of consensuated multi-disciplinary strategies. For instance, given the central role played by the metabolome in relaying information across omic levels, a unified approach to metabolomics studies will be needed to overcome current differences in data types (targeted versus untargeted and liquid chromatography–mass spectrometry (LC–MS) versus gas chromatography–mass spectrometry (GC–MS)) or origin of the specimens (blood/serum, urine or feces). Phenotyping behavorial and GI symptoms in children with ASD is another issue that is still far from being resolved, making it further challenging to stratify patient cohorts. Issues of timescales—from the molecular to the behavioral—need to be harmonized in statistically relevant ways to allow for proper causality inference. Finally, using appropriate statistical methodologies for identifying potential causal relationships will be critical to ensure the success of the proposed mechanistic studies and of efforts to advance understanding of the role that the microbiome plays in the context of the overall functional architecture of ASD.

## Methods

### Search strategy and inclusion criteria

We performed a systematic search for published and/or publicly deposited or not yet published and/or publicly available human microbiome, metabolome, immunome, transcriptome and autism/ASD datasets in several National Center for Biotechnology Information (NCBI) databases (PubMed, Sequence Read Archive (SRA) and BioProject), UCSD’s MassIVE resource, the PsychENCODE consortium and the American Gut Project and from individual research groups worldwide. About half of the 70+ studies that we identified were already deposited on public data repositories or were made directly available to us by the research groups.

Most studies consisted of heterogenous—no genotype or phenotype stratification—ASD and neurotypical age-matched and sex-matched cohorts and had one or two datasets (microbiome (16S, SMS), metabolome (urine/serum/fecal), immunome (cytokines), transcriptome (RNA-seq), dietary survey and behavioral survey) associated with them, with only a few studies having three or more omic datasets associated with them (Table 1). We adopted a multi-cohort and multi-omics meta-analysis framework that allowed us to combine independent and dependent omic datasets in one overall analysis^19^. In total, we analyzed 528 ASD–control pairs that had either age and sex information or sibling-matching information. To reduce the batch effects and noise associated with primer choice in the 16S datasets, a major confounder in microbiome analyses, we restricted the 16S datasets to include only those targeting the variable region V4 of the bacterial ribosomal RNA, a region exhibiting higher heterogeneity and lower evolution rates than other variable regions^64^. Previous studies showed how primers targeting adjacent regions in the 16S can yield similar composition estimates up to the genus level^65^. Our analysis included 16S datasets obtained targeting the V4 region exclusively, the V3–V4 region or the V4–V5 region.

The final metabolomic meta-analysis that we present here consists of the combined analysis of only four independently pre-processed, normalized and analyzed metabolomic datasets. Despite several more ASD-related datasets being available, the disparity in mass spectrometric technologies used to generate them, which results in the detection of different subsets of metabolites, precluded their side-by-side comparison (Table 1). For example, targeted mass spectrometry enables the precise determination of concentrations for a finite number of metabolites, whereas untargeted mass spectrometry detects up to two or three orders of magnitude more metabolites but is compositional in nature and, thus, does not yield absolute abundances. Furthermore, batch effects due to sample processing, such as differences in reagents, sample storage and mass spectrometry instruments, can introduce unwanted variation in both the abundances and the detected molecular features^66^. One additional obstacle that we encountered was the proprietary nature of many of the metabolomic datasets that made it impossible to access the raw data and run standardized workflows.

Of the 40 transcriptomic datasets that were available in recount3 (ref. ^67^), the vast majority were obtained from studies with model animals, and only four of them had been obtained from postmortem processing of brain samples from autistic and neurotypical individuals. These four datasets collected different brain tissue types, including from the amygdala, the prefrontal cortex, the anterior cingulate and the dorsolateral prefrontal cortex.

#### Martin-Brevet et al. cohort

Data from Martin-Brevet et al.^38^ were acquired from two different cohorts: one from the Simons Variation in Individuals Project, consisting mostly of families from the United States. From this cohort, there are 24 individuals with the 16p11.2 deletion and 24 corresponding siblings from the same family who do not carry the deletion; and a second cohort consisting of individuals from the European 16p11.2 consortium (24 deletion carriers and 24 familial controls). More exact information about this cohort was previously published^38^. Deletion carriers were ascertained regardless of age or clinical diagnosis. DNA was extracted from stool samples, and 16S sequencing was performed using primers to the V4 region.

### Data processing

We constructed matched reference databases for 16S and SMS data analyses. The Web of Life 2 (WoL2) reference genome database contains 15,953 bacterial and archaeal genomes sampled from the NCBI to maximize representation of biodiversity. It is a major upgrade from WoL (10,575 genomes). A reference phylogeny was reconstructed based on 387 universal marker genes using uDance, a novel phylogenomic inference workflow employing a divide-and-conquer method. Taxonomic assignments of the genomes were based on Genome Taxonomy Database (GTDB) r207 and curated according to the phylogeny using tax2tree. The Greengenes2 reference 16S rRNA database was constructed based on the WoL2 whole-genome phylogeny and updated with full-length 16S rRNA sequences from the Living Tree Project and 16S from high-quality bacterial operons, using uDance to revise the topology. Into this backbone, we inserted all 16S V4 amplicon sequencing variants from public and private samples Qiita using DEPP. A taxonomy based on GTDB r207, expanded with lineages from the Living Tree Project not present in GTDB, was decorated onto the phylogeny using tax2tree. Full details behind the construction of WoL and Greengenes2 can be found in Usyk et al.^32^.

The 16S amplicon and shotgun metagenomics samples were downloaded from the SRA. The 16S amplicon samples were processed using Deblur and subsequently mapped to Greenegenes2 using Vsearch with qiime2 (ref. ^68^). Shotgun metagenomics samples were mapped to bacterial whole genomes captured in the WoL2 using Bowtie2 followed by Woltka^69^. Viral abundances were extracted from shotgun metagenomics samples using GPD and BWA. RNA expression data were obtained directly from recount3 (ref. ^67^); the four metabolomics datasets were provided by the authors.

To enable age and sex matching, a bipartite matching between individuals with ASD and neurotypical individuals was performed using age and sex covariates. This approach has been shown to be optimal for case–control matching^70^. Individuals who could not be matched were excluded from the meta-analysis. Among the 16S and SMS datasets, there were multiple longitudinal datasets. To integrate these datasets into the cross-sectional analysis, we picked only the first timepoint for each individual.

### Differential ranking analysis

One of the most common approaches to evaluating microbiome and other omic studies consists of determining differences in the abundances of microbial taxa, human metabolites or other omic features between cases and controls. Such differential abundance analysis is typically performed by computing the log fold changes between the case and control groups^21^. However, confounders, such as sex-related, age-related and geography-related batch effects, compositionality, high dimensionality, overdispersion and sparsity, prevented a reliable estimation of differential abundances and, thus, compromised the side-by-side comparison of these differential abundances across studies in the manner of a traditional meta-analysis. Here, we set out to overcome these inherent limitations of traditional meta-analyses by developing a generalizable approach for controlling for select confounders that would help reveal a comprehensive picture of ASD-specific omic signals.

To minimize confounder effects, we developed a Bayesian differential ranking algorithm that uses bipartite matching to optimize the age-based and sex-based pairing of ASD and control individuals within each dataset. This approach helped control for potential age and sex confounders while also minimizing batch effects, such as sample collection method, sample processing protocol, different primers and geographical provenance^71^. Our approach could do this by leveraging recent insights into the multiplicative nature of protocol biases^18^. Fold change calculations can be designed to be robust to bias induced by protocols, provided that the fold changes are being computed only on samples processed under the same protocol. Similar observations have been made about biases induced by differences in polymerase chain reaction (PCR) primers, with abundance-based beta diversity metrics being robust to primer biases, as long as comparisons are confined to datasets generated with the same protocol^71^. We extended this strategy to handle age and sex matching, taking advantage of the fact that most of the cohorts that we analyzed selected their participants to be age and sex matched. Most of the case–control pairings in the 16S and SMS datasets were within 1 year apart, providing an opportunity to remove age-related confounders in downstream analyses. Our Bayesian models were fitted via Markov chain Monte Carlo (MCMC) using Stan^72^. Conceptually, this allowed us to compute log fold change differences of microbes between age-matched and sex-matched individuals, but, because we did not have absolute abundance information, we could estimate this log fold change only up to a constant^73^ (Supplementary Information).

To determine how sensitive our proposed differential abundance strategy was to sequencing depth, we conducted a rarefaction benchmark in addition to a simulation benchmark. When comparing unrarefied data (with mean sequencing depth greater than 200,000 reads per sample) and sequencing count data rarefied down to 9,000 reads per sample from the 16S cross-sectional cohort data, we still see strong agreement between the unrarefied log fold changes and the rarefied log fold changes (Extended Data Fig. 2e). This supported the theoretical evidence that our differential abundance method was scale equivariant and that changes in sequencing depth would not markedly affect the mean log fold change estimates.

This was further validated in our simulation benchmarks, where we showed that our model could capture the ground truth log fold changes based on 16S differentials from the age-matched and sex-matched cohort (Extended Data Fig. 2f). We compared our proposed age-matched and sex-matched differential ranking method to ANCOM-BC and our differential abundance method without age and sex matching (which we will refer to as group-averaged differential ranking) (Extended Data Fig. 2i–k) to showcase the differences between these methods. This benchmark was performed using data-driven simulations derived from the 16S cohort analysis. For the side-by-side comparison, we ran three different configurations of ANCOM-BC: (1) case–control differences only [‘formula=disease status’]; (2) case–control differences adjusted by age and sex confounders [‘formula=disease status + age + sex’]; and (3) case–control differences by age and sex matching [‘formula=disease status + (disease status–age sex matching IDs)’]. The first configuration provided a direct comparator to our ‘standard group-averaged differential ranking’, and the third configuration provided the most direct comparator to our ‘sex- and age-matched differential ranking’. None of the three ANCOM-BC models could recover the ground truth log fold changes in our simulations (*r* = 0.38, 0.37 and 0.39 for implementations 1, 2 and 3, respectively), whereas both the ‘standard group-averaged differential ranking’ and the ‘sex- and age-matched differential ranking’ models were able to recover the ground truth (*r* = 0.64 and 0.79, respectively). Ultimately, this illustrates how our method could account for age and sex matching and perform as expected if the assumptions were satisfied.

**Extended Data Fig. 6:**
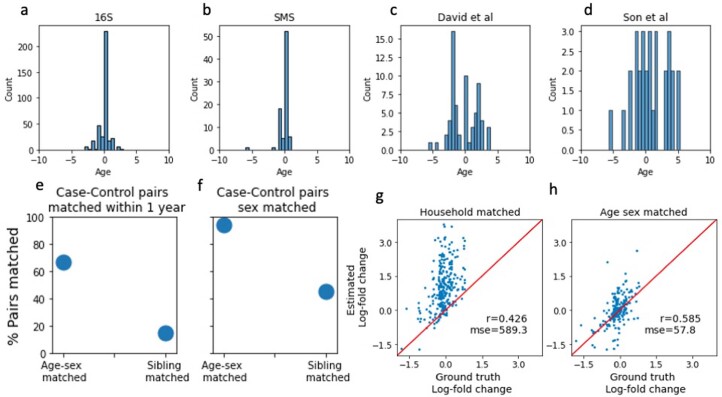
Age differences between case-control matchings. (a) 16S age-sex matched dataset, (b) the SMS age-sex matched dataset, (c) the David et al household matched dataset (16S)^37^ (d) the Son et al household matched dataset (16S)^36^ all datasets, the age of the control subject is subtracted from the age of the corresponding matched ASD subject. Neither David et al^37^ or Son et al^36^ showed a statistical difference between ages across households. (e) Estimated microbial log-fold changes compared to ground truth microbial log-fold changes in household matched simulation. (f) Estimated microbial log-fold changes compared to ground truth microbial log-fold changes in age-sex matched simulation. (g) Percentage of case-control pairs that are within 1 year in the age-sex matched dataset and the sibling matched dataset. (h) Percentage of case-control pairs that are have the same gender in the age-sex matched dataset and the sibling matched dataset.

**Extended Data Fig. 7:**
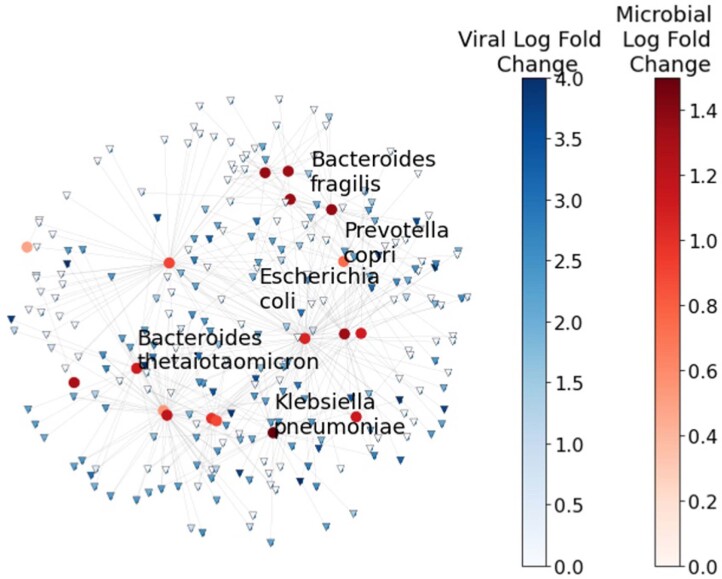
Microbe-viral co-occurrence network estimated using MMvec. Microbes are colored red and viruses are colored blue. Edges are drawn between microbes and viruses if they are highly co-occurring and the interaction was annotated in GPD.

**Extended Data Fig. 8:**
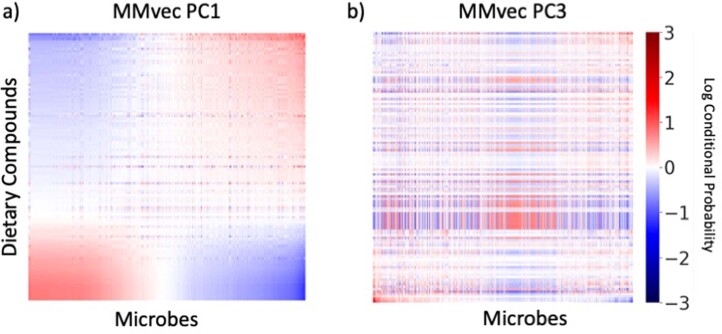
Microbe-diet co-occurrences. Microbe-diet co-occurrence heatmaps sorted by the (a) first and (b) third principal components estimated from MMvec.

**Extended Data Fig. 9:**
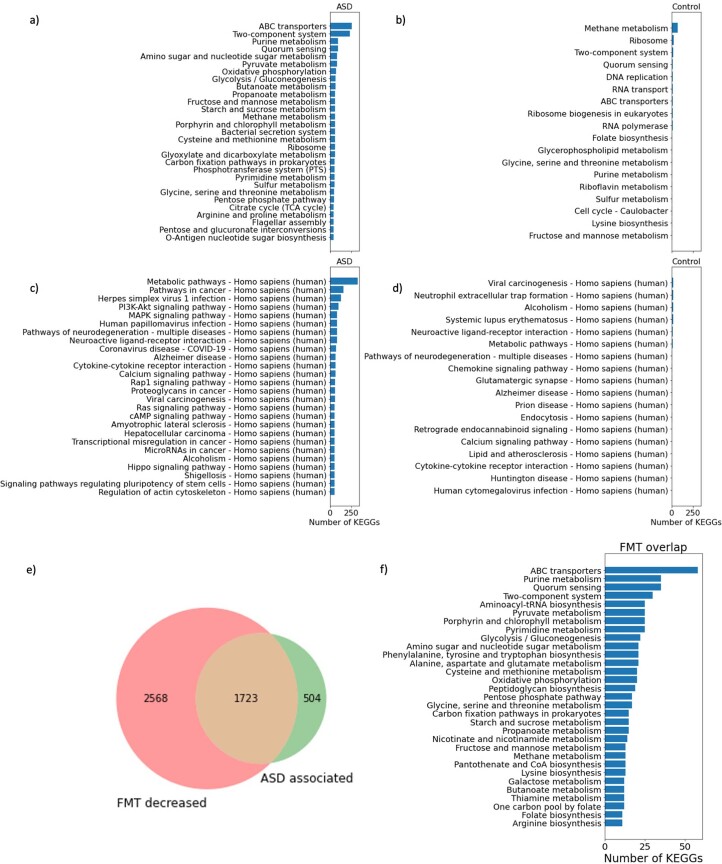
Distribution of pathways in ASD and control-associated genes detected in SMS and RNAseq data. (a-b) Breakdown of pathways in SMS data that are associated with ASD and neurotypical controls. (c-d) Breakdown of pathways in RNAseq data that are associated with ASD and neurotypical controls. e) Overlap of ASD associated KEGG enzymes derived from the multi-cohort cross-sectional analysis and KEGG enzymes that are found to be present in the microbes that decreased in the Kang et al FMT study. f) Pathway break down of KEGG enyzmes found in both the Kang et al FMT study and ASD children in the multi-cohort cross-sectional analysis. Only microbes that were also found in the SMS data were considered in the Kang et al study.

Similar to other simulation-based benchmarks, this is not a rigorous benchmark showcasing the improved performance of our method; rather, it is showcasing how all three methods have different assumptions. To determine in which biological scenarios age and sex matching could be more informative than household matching, we generated another simulation incorporating both a household confounder and an age confounder. The subject ages and age differences were sampled from the age distribution observed in David et al.^37^. Similarly to our previous simulations, we simulated the ground truth log fold changes using the model from the 16S cohort analysis. Here, we observed that, with a sufficiently large age confounder, the household matching estimated log fold changes with a noticeably large bias (mean squared error = 589.3) (Extended Data Fig. 6e). In contrast, although age and sex matching did not precisely estimate the ground truth log fold changes, we observed a 10×-fold reduction in bias (mean squared error = 57.8) (Extended Data Fig. 6f). This simulation also showcased how cohort randomization may play a role in mitigating the bias introduced by age confounding, at the expense of increased variance of the estimator.

To determine how sensitive our proposed differential abundance strategy is to batch effects, we computed the log fold changes between two samples, ‘sample4’ and ‘sample6’, from the MBQC study^31^ for each processing laboratory. These samples were replicated and processed by multiple laboratories, providing an experimental setup for validating batch removal methods. Bray–Curtis PCoA shows a weak separation in sample name but a strong separation due to batch effects induced by differences in processing protocols. However, when we compare the log fold changes for each processing laboratory, we see strong agreement (*r* > 0.5, *P* < < 0.05) (Extended Data Fig. 6m), which supports the claim of McLaren et al.^18^ that within-study fold change calculations are insensitive to batch effect, as long as the processing protocol is consistent within the study.

To determine if there was a significant difference between the age-matched and sex-matched pairs, we constructed an effect size metric using our model’s uncertainty estimation (see Supplementary Methods for more details). A global model for each data type—16S, SMS and RNA-seq—was used to determine if there was a significant difference between age-matched and sex-matched case–control pairs across each datatype. When we evaluated our Bayesian model fit on the 16S, SMS and RNA-seq datasets, our model fits achieved Rhat values below 1.1 and ESS values above 300, indicating that the draws from the posterior distribution are reliable.

The age-matched and sex-matched classifiers were constructed to build a classifier that generalizes across cohorts, identifying microbes that consistently differentiate between age-matched and sex-matched case–control pairs. To build age-matched and sex-matched classifiers, within each age-matched and sex-matched 16S cohort, we fitted our Bayesian model and assigned taxa into three groups: ASD-associated, Control-associated and Neutral. A taxon is assigned to the ASD-associated group if 70% of their posterior samples are greater than 0; a taxon is assigned to the Control-associated group if 70% of their posterior samples is less than 0. The remaining taxa are assigned to the Neutral group. After assigning taxa to each group, for each sample, a single log ratio, or balance^15^, is computed by taking the geometric mean of all of the taxon abundances within each group. To create a single log ratio that generalizes across cohorts, we assigned taxa to the ASD-associated group if it appears to be ASD associated in at least two studies. The same procedure is applied to the Control-associated group. The differences of these log ratios across the case–control pairs are shown for the age-matched and sex-matched cohorts in Fig. 2c. Although we did not apply this approach to the shotgun metagenomics datasets, we showed that the log ratios constructed from the 16S datasets also separated more than 70% of the ASD–control samples, serving as an additional cross-validation.

To determine if a microbe in increased or decreased between two groups of samples, a reference frame that identifies which group of microbes is stable is required. To do this using our Bayesian models, the quantiles estimated from the posterior distribution of the log fold change is used. A microbe is said to be significantly increased if the log fold change is greater than 0 in 95% of the posterior samples (5% log fold change quantile > 0). Finally, a microbe is said to be stable if the 90% quantile of the posterior distribution overlaps with 0 and the standard deviation of the posterior distribution is less than 3. Similarly, a microbe is said to be significantly decreased if the log fold change is less than 0 in 95% of the posterior samples (95% log fold change quantile < 0). The reference frame in the FMT analysis used microbes that were identified to be neutral or control associated by the age-matched and sex-matched classifier, with the assumption that the average abundance of these taxa is stable throughout the entire 2-year follow-up study. The FMT analysis used the same matching strategy, but, instead of matching on age and sex, the matchings were performed on the subjects to compare different timepoints. When identifying microbes that are the core microbiome, we focused on taxa that overlapped with 0 and had a posterior standard deviation of less than 3. Similarly, when computing the overlap between the cross-sectional cohort and the microbes depleted after the FMT, we focused on taxa with low uncertainty with a posterior standard deviation of less than 3.

The heat map shown in Fig. 2b displays the log fold changes for each case–control pair. To do this, a robust center log ratio (CLR) transform was performed, and all zeros were imputed to the mean abundance for visualization purposes. The case–control log fold changes were then computed for each case–control pair.

#### Bayesian differential ranking

Conceptually, the goal of a differential analysis is to make a statement about change in abundance for a given feature *i* between conditions A and B by evaluating the following null hypothesis:

$$\frac{{A}_{i}}{{B}_{i}}=1$$

However, most omic datasets do not provide a direct observation of the absolute quantities of *A*_*i*_ and *B*_*i*_, or the total microbial loads ${N}_{{A}_{i}}$ and ${N}_{{B}_{i}}$ but, rather, only an observation of their proportions ${p}_{{A}_{i}}$ and ${p}_{{B}_{i}}$, respectively, within each dataset, which are determined by a bias term, $\frac{{N}_{A}}{{N}_{B}}$. This bias term, given by

$$\frac{{A}_{i}}{{B}_{i}}=\frac{{p}_{{A}_{i}}{N}_{A}}{{p}_{{B}_{i}}{N}_{B}}=\frac{{p}_{{A}_{i}}}{{p}_{{B}_{i}}}\frac{{N}_{A}}{{N}_{B}}$$

results in high FDRs that cannot be adjusted for in models analyzing compositional omics datasets because the overall contribution of *N*_*A*_ and *N*_*B*_ to change cannot be unequivocally quantified^74^. To avoid the total biomass bias without having to resort to performing traditional FDR corrections, we adopted a ranking approach that allowed us to sort omic features by their log fold change values independently of how large their change was in absolute terms^73^. Because the biomass bias impacts every species within a dataset equally, the ranking approach ignores this bias, making the approach scale invariant (Equation 1).

$${{{\rm{rank}}}}\left(\frac{A}{B}\right)={{{\rm{rank}}}}\left(\frac{{p}_{A}{N}_{A}}{{p}_{B}{N}_{B}}\right)={{{\rm{rank}}}}\left(\frac{{p}_{A}}{{p}_{B}}\right)$$

The overall model that we designed consisted of a customized differential abundance tool that leveraged the experimental design of each study included in the analysis to determine study-specific feature perturbation profiles that could then be combined with the normalized perturbation profiles of other studies to perform a global differential perturbation analysis. The overall model had the following structure:

$$\begin{array}{rcl}{y}_{i,\,j}& \sim &{{{\rm{NegativeBinomial}}}}({\lambda }_{i,\,j},{\alpha }_{ij})\\ \log {\lambda }_{i,\,j}&=&\log {N}_{i}+{C}_{k(i),\,j}+{D}_{j}{{{\rm{I}}}}[i=ASD]\end{array}$$

where *y*_*i*, *j*_ denotes the microbial counts in sample *i* of species *j* across *d* species; *λ*_*i*, *j*_,*α*_*ij*_ represents the expected counts for species *j*; sample *i*, *j* represents a microbe-specific overdispersion term; *N*_*i*_ represents sequencing depth (self-normalization and preemptive of rarefaction); *C*_*k*(*i*),*j*_ represents the log proportion of species *j* in the *k*(*i*) control subject (age matched and sex matched); and *D*_*j*_I[*i* = *ASD*] represents the log fold change difference between control and ASD subjects with a corrective function that equals 1 when *i* corresponds to the paired ASD subject and 0 when *i* corresponds to the control subject. Incorporating *N*_*i*_ into the model renders the model self-normalizing and not dependent on rarefaction, and *C*_*k*(*i*),*j*_ incorporates the age-matching and sex-matching component for a given pair *k*. The priors for these variables are given below.

$$\begin{array}{rll}{\alpha }_{ij}&=&\frac{{a}_{0,k(i),\,j}}{{\lambda }_{ij}}+{a}_{1,k(i),\,j}+{\beta }_{p}\quad {a}_{0} \sim {{{\rm{LogNormal}}}}(0,1)\\ && {a}_{1} \sim {{{\rm{LogNormal}}}}(\log (10),0.1)\\ {\beta }_{p} &\sim& {{{\rm{Normal}}}}({\beta }_{\mu },{\beta }_{\sigma })\quad{\beta }_{\mu } \sim {{{\rm{Normal}}}}(0,3)\\ && {\beta }_{\sigma } \sim {{{\rm{LogNormal}}}}(\log (0.1),0.1)\\ {C}_{k(i),\,j} &\sim & {{{\rm{Normal}}}}({C}_{{\mu }_{j}},{C}_{{\sigma }_{j}})\quad{C}_{{\mu }_{j}} \sim {{{\rm{Normal}}}}\left(\frac{1}{d},3\right)\\ && {C}_{{\sigma }_{j}} \sim {{{\rm{Normal}}}}\left(\frac{1}{d},1\right)\\ {D}_{j} &\sim& {{{\rm{Normal}}}}(0,3)\end{array}$$

Here, the overdispersion parameters are estimated for each microbe, for each batch and for the ASD and control groups. This approach is adapted from DESeq2, allowing for the overdispersion to be modeled by both linear and quadratic terms with respect to the abundance. Furthermore, this parameterization does allow for a compositional interpretation owing to the following rationale. The Poisson distribution with an offset term is known to approximate the multi-nomial distribution. Furthermore, the negative binomial can be re-parameterized as a gamma–Poisson distribution, allowing for overdispersion modeling by breaking the mean–variance relationship inherent in the Poisson distribution.

The age-matched and sex-matched differential abundance has a similar methodology to paired tests, such as the paired *t*-test and the Wilcoxon test. To this end, we also used this differential abundance methodology to analyze the FMT dataset. Here, instead of matching pairs of subjects, we matched pairs of timepoints and computed the differential abundance across each pair of timepoints. To make these differentials comparable, a common set of taxa that was detected to be associated with controls was selected. Specifically, taxa that had a log fold change less than 0 in the cross-sectional cohort were assigned to this reference set. The estimated log fold changes were adjusted by centering around the mean log fold in the reference dataset as follows:

$${{{{\boldsymbol{D}}}}}^{* }={{{\boldsymbol{D}}}}-{\bar{{{{\boldsymbol{D}}}}}}_{R}$$

where ${\bar{{{{\boldsymbol{D}}}}}}_{R}$ denotes the mean of the log fold changes of the reference taxa, and D^*^ represents the recentered log fold changes. By doing this, all timepoints will have the same reference and will be more directly comparable.

One of the advantages of the above model is that it will cancel out any multiplicative batch effect, such as PCR amplification bias, with no impact on *j*. This is because *D* is computed only within cohorts, and, as a result, cohort-specific batch effects are mitigated. Another advantage of the proposed model is that negative binomial models can be fitted independently for each microbe; as a result, the log fold change estimates for one microbe will not affect the estimates of other microbes. This can be a benefit, because these models will be agnostic to the choice of filtering criteria—filtering certain microbes will not affect the log fold change estimates of the remaining microbes. Furthermore, this differential abundance model can be applied to different types of omics data. Moreover, because we built the differential ranking model in a Bayesian environment, we were able to fit the model using an MCMC approach to estimate uncertainty by sampling the resulting posterior distributions.

For example, to make a statement about the value of an estimated posterior probability distribution *p*(*D*∣ *y*), we could compute an average value using the following approximation:

$${\mathbb{E}}[D]\approx \frac{1}{m}\mathop{\sum }\limits_{i=0}^{m}{\hat{D}}_{i}\quad {\hat{D}}_{i} \sim p(D| \,y)$$

Using this classic application of MCMC sampling in which *N* samples of *i* are drawn from the posterior distribution *p*(*D*∣*y*), we were able to approximate the true mean of the posterior differential abundance distributions and the corresponding effect sizes. With this, we can compute an effect size metric that determines if there is any global difference detected. This metric is analogous to PERMANOVA but one that computes this from log fold changes using the age-matched and sex-matched design. The effect size *E* is measured as follows:

$$E=\frac{| | {\mu }_{D}| {| }_{2}}{{r}_{D}},\quad {\mu }_{D}=\frac{1}{m}\mathop{\sum }\limits_{i=0}^{m}{\hat{D}}_{i}\quad {r}_{D}=\mathop{\max }\limits_{{\hat{D}}_{i} \sim p(D| y)}\parallel | {\hat{D}}_{i}-{\mu }_{D}\parallel {| }_{2}$$

where *μ*_*D*_ is the mean of the posterior distribution, and *r*_*D*_ represents the radius of a sphere that contains all of the samples from the posterior distribution. If the effect size is greater than 1, that means that 0 is not included in the posterior distribution, and the difference is significant. Bayesian *P* values are computed from the number of draws of ${\hat{D}}_{i}$ that were simulated from the posterior distribution *p*(*D*∣*y*). For instance, if 100 draws are sampled from the posterior distribution, and 0 is not within the sphere estimated from those 100 draws, then we say that the posterior distribution is significantly not overlapping with 0 with *P* < 0.01.

### Other methods

We fitted gradient boosting classifiers on 10 16S datasets and on three SMS datasets using q2-sample-classifier^68^. We randomly split the samples into 80/20 training and test splits, performed a fivefold cross-validation on the training datasets to obtain optimal model parameters and computed predictions on the held-out test dataset. PERMANOVA with Bray–Curtis distances was used to determine if confounding variation due to household, age and sex was statistically significant in the sibling cohorts.

We used MMvec^73^ to perform the diet–microbe co-occurrence analysis. Here, microbes were used to predict dietary intake. This analysis enabled the estimation of conditional probabilities, namely the probability of observing a dietary compound given that the microbe was already observed. To estimate these conditional probabilities, MMvec performs a matrix factorization, identifying the factors that explain the most information in these interactions. We compared the MMvec microbial factors against the cross-sectional log fold changes. We then compared the MMvec dietary factors against *t*-statistics that measure the differences in dietary compounds between children with ASD and neurotypical children.

To identify candidate viral–microbe interactions, we ran MMvec on each of the SMS datasets. We then pulled out the top co-occurring viral taxa for each microbe that had a conditional log probability greater than 1, amounting to 78,580 microbe–viral interactions. Then, we filtered out the microbe–viral interactions that were not present in the Gut Phage Database (GPD)^44^, leaving 31,276 microbial–viral interactions. The microbe–viral interactions estimated by Dan et al.^28^ and Wang et al.^33^ were weakly generalizable (*Q*^2^ =0.0036 > 0 and *Q*^2^ =0.0114 > 0). However, the microbe–viral interactions estimated from Averina et al.^34^ were similar to random chance (*Q*^2^ = −0.005).

We used Songbird^15^ to perform the cytokine−microbe analysis via a multinomial regression that used the cytokines to predict microbial abundances. We reported biased microbial log fold changes with respect to cytokine concentration differences. Pearson correlation was used to determine the agreement between the 16S cross-sectional microbial differentials and the microbe−cytokine differentials. To directly link these microbial abundances to the cytokine concentrations, we computed log ratios, or balances, of microbes for each sample. For example, for IL-6, the numerator consisted of the top 50 microbes that are estimated to increase the most in abundance when IL-6 concentration increased, and the denominator consisted of the bottom 50 microbes that are estimated to be the most decreased when IL-6 concentration increases. Once these partitions are defined, the balances for each sample are computed by taking the log ratio of the average abundance of the numerator group and the denominator group^15^. Pearson correlation between these balances and the cytokine concentrations is then computed to measure the agreement between the microbial abundances and the cytokine concentrations.

To identify key microbial genes, we performed a comparative genomic analysis in which we binned the microbial genomes into those associated with ASD subjects and those associated with control subjects in the shotgun metagenomics data. We focused on microbes that are strongly associated with ASD, specifically those that are significantly greater than 10% of taxa that are estimated to be enriched in ASD. Using a binomial test, we were able to determine if a particular gene was more commonly observed in ASD-associated microbes than by random chance. Altogether, we identified 2,176 statistically significant microbial genes that differentiated ASD-associated microbial genomes from neurotypical-associated microbial genomes. Similarly, we identified 1,570 human transcripts that were differentially expressed between ASD and neurotypical subjects. Significant microbial genes and RNA transcripts were subsequently mapped to KEGG pathways. To directly compare the two contrasting omics levels and gauge metabolic similarity, we retrieved all the molecules involved in both the microbial and human pathways and calculated their intersection. Because the metabolomics datasets are not discrete values like sequencing count data, we additive log ratio (ALR) transformed the metabolomics datasets using the reference frames highlighted in the original papers. We then performed Wilcoxon tests on age-matched and sex-matched metabolomics samples within each cohort separately. Although our analysis revealed multiple metabolites that were below the 0.05 *P* value threshold, none of these metabolites passed the FDR-corrected threshold.

### Reporting summary

Further information on research design is available in the Nature Portfolio Reporting Summary linked to this article.

## Online content

Any methods, additional references, Nature Portfolio reporting summaries, source data, extended data, supplementary information, acknowledgements, peer review information; details of author contributions and competing interests; and statements of data and code availability are available at 10.1038/s41593-023-01361-0.

## Supplementary information

Supplementary Information Supplementary Tables 1–3.

Reporting Summary

Supplementary Tables 4–16 Table S4: Table of statistics for 16S differentials, including mean log fold change, standard deviation log fold change, 90% credible intervals and taxonomy for each microbe. Table S5: Table of statistics for SMS differentials, including mean log fold change, standard deviation log fold change, 90% credible intervals and taxonomy for each microbe. Table S6: Table of statistics for RNA-seq differentials, including mean log fold change, standard deviation log fold change and 90% credible intervals for each transcript. Table S7: Table of statistics for viral differentials, including mean log fold change, standard deviation log fold change and 90% credible intervals for each virus. Table S8: PERMANOVA breakdown of sibling-matched cohorts looking at the confounding variation due to age, sex and household. Table S9: Microbial log fold changes due to cytokine differences, including mean log fold change for each cytokine. Table S10: Microbe virus co-occurrences estimated by MMvec, where entries represent the centered log probability of a microbe and a virus both present for a given sample. The first two columns yield the *t*-statistic and a *P* value measuring the difference between case and control dietary preferences. Table S11: A list of genes and their associated KEGG pathways that were determined to be statistically abundant in ASD-associated microbial genomes using a one-sided binomial test corrected for multiple comparisons. Table S12: A list of genes and their associated KEGG pathways that were determined to be statistically expressed in humans using a one-sided binomial test corrected for multiple comparisons. Table S13: A list of microbial pathways that were both statistically abundant in ASD-associated microbial genomes and present in GBMs. Table S14: A list of paired microbe and human pathways in addition to the number of overlapping metabolites. Table S15: Microbe diet co-occurrences estimated by MMvec, where entries represent the centered log probability of a microbe and a dietary compound both present for a given subject. Table S16: Microbial log fold changes between paired timepoints across all of the individuals in the FMT study. The reported log fold change was calculated by using the reference frame estimated in the cross-sectional analysis.

## Figures and Tables

**Extended Data Fig. 5:**
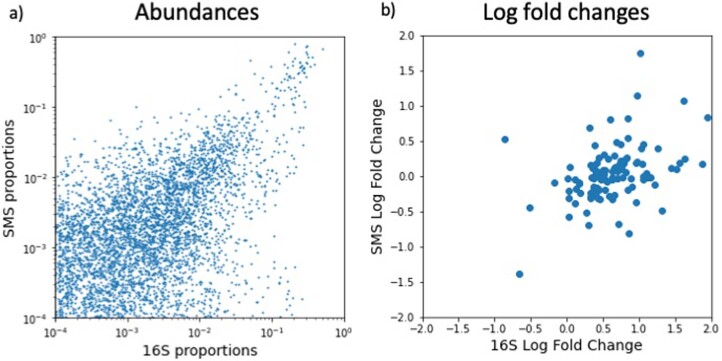
Comparison of log-fold changes computed from 16S and SMS. (a) Comparison of taxa proportions across all 16S and SMS samples from Dan et al^28^ the cross-sectional datasets after mapping to Greengenes2. (b) Comparison of differentials obtained from 16S and SMS on the same samples from Dan et al across taxa observed in both datasets. Only log-fold changes with high confidence (std < 0.5) are shown here.

## Data Availability

This study is based on previously published 16S^22–25,27–29,37,38,52,36^, metagenomics^28,33,34^, RNA-seq^59–62^ and metabolomics^48–50^ data. (The 16S sequencing data in Martin-Brevet et al.^38^ is available under accession number ERP147524. All processed datasets and harmonized metadata are available on Zenodo at 10.5281/zenodo.7877350 as well as on Github at https://github.com/mortonjt/asd_multiomics_analyses.

## Code availability

Software implementation of our Bayesian age-matched and sex-matched differential ranking algorithm can be found at https://github.com/flatironinstitute/q2-matchmaker. Our group-averaged differential ranking algorithm can be found at https://github.com/mortonjt/q2-differential. Finally, our analysis scripts can be found at https://github.com/mortonjt/asd_multiomics_analyses. We would like to acknowledge Matplotlib, Seaborn, Scipy, Numpy, Xarray, Arviz Scikit-learn, biom-format and Scikit-bio for providing the software foundation that this work was built upon.
