# Supplementary material for: Multi-level analysis of the gut–brain axis shows autism spectrum disorder-associated molecular and microbial profiles
Source: Nat Neurosci. 2023 Jun 26;26(7):1208–17. doi: 10.1038/s41593-023-01361-0 (PMC10322709; doi:10.1038/s41593-023-01361-0)
Supplement: Supplementary file 1 — Supplementary Tables 1–3. [file 41593_2023_1361_MOESM1_ESM.pdf]

nature neuroscience

Article

https://doi.org/10.1038/s41593-023-01361-0

Multi-level analysis of the gut–brain axis shows autism spectrum disorder-associated molecular and microbial profiles

In the format provided by the authors and unedited

Cytokine Assignment	Case-Control Assignment	Microbe
Depleted wrt IL-6	ASD associated	*Anaerobutyricum hallii*
		Ruminococcus_D bicirculans
		Anaerostipes hadrus
		Ruminococcus_A sp003011855
		Blautia_A obeum
		Akkermansia muciniphila_D
		Faecalibacterium prausnitzii_C
		Streptococcus vestibularis
		Dorea_A formicigenerans
		Mediterraneibacter_A faecis
		Coprococcus_A catus
		Roseburia inulinivorans
Enriched wrt IL-6	ASD associated	*Anaeromassilibacillus sp001305115*
		Acetatifactor sp900066565
		Alistipes_A communis
		CAG-269 sp000431335
		Phocaeicola vulgatus
		Faecalibacterium prausnitzii_C
		Dysosmobacter sp000403435
		Parabacteroides_B distasonis
		Schaedlerella sp900066545
		Alistipes_A finegoldii
		Butyrivibrio_A crossotus
Depleted wrt IL-6	Neurotypical associated	*Desulfovibrio_R fairfieldensis*
Depleted wrt TGF-β	ASD associated	*Bacteroides_E thetaiotaomicron*
		Enterocloster sp000431375
		Firm-11 sp900540045
		Blautia_A obeum
		Lachnoclostridium_B sp900066555
		CAG-269 sp000431335
Enriched wrt TGF-β	ASD associated	*Eubacterium_I ramulus*
		Faecalibacterium prausnitzii_C
		Ruminococcus_D bicirculans
		Schaedlerella sp900066545
		Dysosmobacter sp000403435
		Enterocloster sp000431375
		UMGS1375 sp900066615
		Alistipes_A finegoldii
		Butyrivibrio_A crossotus
		Acetatifactor sp900066565
		Parabacteroides_B distasonis

Table S1: Breakdown of cytokine differentials for microbes with annotated taxonomies. Microbes are assigned to “Depleted wrt IL-6” if they are amongst the top 25% of microbes that are the most decreased with respect to IL-6 concentration. Microbes are assigned to the “ASD associated group” if the 5% quantile of their posterior distribution is greater than 0. Similarly, microbes are assigned to “Enriched wrt IL-6” if they are amongst the top 25% of microbes that are the most increased with respect to IL-6 concentration. Microbes are assigned to the “Neurotypical associated group” if the 95% quantile of their posterior distribution is less than 0. The same process is applied to TGF-β differentials.

Dietary Assignment	Case-Control Assignment	Microbe
AA deficient	Neurotypical associated associated	*Negativibacillus massiliensis*
AA deficient	ASD associated	*P. copri*
Not AA deficient	Neurotypical associated	*Parabacteroides_B distasonis*<br>*Alistipes_A onderdonkii*
Not AA deficient	ASD associated	*Bifidobacterium longum*<br>*Lactobacillus casei*<br>*Lactococcus_A lactis*<br>*Eubacterium_O maltosivorans*

Table S2: Breakdown of microbe-diet co-occurrence analysis for microbes with annotated taxonomies. Microbes are assigned to "AA deficient" if they are amongst the top 25% of microbes that are the most decreased with respect to MMvec principal component 3, which was correlated with amino acid deficiency. Microbes are assigned to the "ASD associated group" if the 5% quantile of their posterior distribution is greater than 0. Similarly, microbes are assigned to "Not AA deficient" if they are amongst the top 25% of microbes that are the most increased with respect to MMvec principal component 3. Microbes are assigned to the "Neurotypical associated group" if the 95% quantile of their posterior distribution is less than 0.

FMT assignment	Microbe
Stable	*Prevotella corporis*<br>*Prevotella timonensis*<br>*Prevotella copri*<br>*Bifidobacterium merycicum*<br>*Bifidobacterium faecale*<br>*Bifidobacterium callitrichidarum*<br>*Bifidobacterium bifidum*<br>*Bacteroides_E thetaiotaomicron*<br>*Bacteroides_E fragilis*<br>*Bacteroides_E cellulosilyticus*<br>*Bacteroides_E stercoris*<br>*Bacteroides_E nordii*<br>*Desulfovibrio_R desulfuricans_A*
Enriched	*Bacteroides_E intestinalis*<br>*Muribaculum sp002492595*<br>*Prevotella sp003447235*<br>*Sutterella wadsworthensis_A*<br>*Desulfovibrio_R piger_A*<br>*Coprobacter secundus*<br>*Parabacteroides_B goldsteinii*

Table S3: Breakdown of microbes altered 100 weeks after the FMT. Out of 305 microbes that were detected to be roughly equal to their initial abundances before the FMT, 13 of these microbes fall within the *Prevotella*, *Bifidobacterium*, *Bacteroides* and *Desulfovibrio* genera. 7 out of 8 genera that were enriched 100 weeks after the FMT had taxonomic annotations.
