# Supplementary material for: Multi-level analysis of the gut–brain axis shows autism spectrum disorder-associated molecular and microbial profiles
Source: Nat Neurosci. 2023 Jun 26;26(7):1208–17. doi: 10.1038/s41593-023-01361-0 (PMC10322709; doi:10.1038/s41593-023-01361-0)
Supplement: Supplementary file 2 — Reporting Summary [file 41593_2023_1361_MOESM2_ESM.pdf]

nature portfolio

Corresponding author(s): Gaspar Taroncher-Oldenburg
James T. Morton

Last updated by author(s): 4/28/2023

Reporting Summary

Nature Portfolio wishes to improve the reproducibility of the work that we publish. This form provides structure for consistency and transparency in reporting. For further information on Nature Portfolio policies, see our Editorial Policies and the Editorial Policy Checklist.

Statistics

For all statistical analyses, confirm that the following items are present in the figure legend, table legend, main text, or Methods section.

n/a	Confirmed	
☐	☒	The exact sample size (*n*) for each experimental group/condition, given as a discrete number and unit of measurement
☒	☐	A statement on whether measurements were taken from distinct samples or whether the same sample was measured repeatedly
☐	☒	The statistical test(s) used AND whether they are one- or two-sided<br>*Only common tests should be described solely by name; describe more complex techniques in the Methods section.*
☐	☒	A description of all covariates tested
☐	☒	A description of any assumptions or corrections, such as tests of normality and adjustment for multiple comparisons
☐	☒	A full description of the statistical parameters including central tendency (e.g. means) or other basic estimates (e.g. regression coefficient) AND variation (e.g. standard deviation) or associated estimates of uncertainty (e.g. confidence intervals)
☐	☒	For null hypothesis testing, the test statistic (e.g. *F*, *t*, *r*) with confidence intervals, effect sizes, degrees of freedom and *P* value noted<br>*Give P values as exact values whenever suitable.*
☐	☒	For Bayesian analysis, information on the choice of priors and Markov chain Monte Carlo settings
☐	☒	For hierarchical and complex designs, identification of the appropriate level for tests and full reporting of outcomes
☐	☒	Estimates of effect sizes (e.g. Cohen's *d*, Pearson's *r*), indicating how they were calculated

Our web collection on statistics for biologists contains articles on many of the points above.

Software and code

Policy information about availability of computer code

Data collection	Sequence data was retrieved from the Sequence Read Archive (SRA). Metabolomics data, dietary surveys, and cytokine data were collected directly from the co-authors.
Data analysis	Software implementation of our Bayesian age-sex matched differential ranking algorithm can be found at https://github.com/flatironinstitute/q2-matchmaker<br>Our group-averaged differential ranking algorithm can be found at\\ https://github.com/mortonjt/q2-differential<br>Finally, our analysis scripts can be found at<br>https://github.com/mortonjt/asd_multiomics_analyses<br>We want to acknowledge Matplotlib , Seaborn, Scipy , Numpy , Xarray , Arviz, Scikit-learn , biom-format and Scikit-bio for providing the software foundation that this work was built upon.

For manuscripts utilizing custom algorithms or software that are central to the research but not yet described in published literature, software must be made available to editors and reviewers. We strongly encourage code deposition in a community repository (e.g. GitHub). See the Nature Portfolio guidelines for submitting code & software for further information.

Data

Policy information about availability of data

All manuscripts must include a data availability statement. This statement should provide the following information, where applicable:

- Accession codes, unique identifiers, or web links for publicly available datasets
- A description of any restrictions on data availability
- For clinical datasets or third party data, please ensure that the statement adheres to our policy

All processed datasets and harmonized metadata is available at https://github.com/mortonjt/asd_multiomics_analyses

Research involving human participants, their data, or biological material

Policy information about studies with human participants or human data. See also policy information about sex, gender (identity/presentation), and sexual orientation and race, ethnicity and racism.

Reporting on sex and gender	Sex and gender information was collected from published data.
Reporting on race, ethnicity, or other socially relevant groupings	*Please specify the socially constructed or socially relevant categorization variable(s) used in your manuscript and explain why they were used. Please note that such variables should not be used as proxies for other socially constructed/relevant variables (for example, race or ethnicity should not be used as a proxy for socioeconomic status).<br>Provide clear definitions of the relevant terms used, how they were provided (by the participants/respondents, the researchers, or third parties), and the method(s) used to classify people into the different categories (e.g. self-report, census or administrative data, social media data, etc.)<br>Please provide details about how you controlled for confounding variables in your analyses.*
Population characteristics	Most of the samples were from children under 16 years old, with the exception of the RNAseq data and the data from the 16p cohort.
Recruitment	N/A
Ethics oversight	*Identify the organization(s) that approved the study protocol.*

Note that full information on the approval of the study protocol must also be provided in the manuscript.

Field-specific reporting

Please select the one below that is the best fit for your research. If you are not sure, read the appropriate sections before making your selection.

☒ Life sciences ☐ Behavioural & social sciences ☐ Ecological, evolutionary & environmental sciences

For a reference copy of the document with all sections, see nature.com/documents/nr-reporting-summary-flat.pdf

Life sciences study design

All studies must disclose on these points even when the disclosure is negative.

Sample size	We searched all publicly available autism studies that had fecal metagenomics data.
Data exclusions	We did not include non-fecal samples or samples from mothers, because these inclusion criteria were not relevant for our study. These exclusion criteria were pre-established.
Replication	The reported results are reproducible on our computing cluster environment. We ran fitted these models up to 6 times per dataset. Reproducing these results will require 100s of CPUs and several days of model fitting.
Randomization	An age-sex matching algorithm was applied to match ASD children to neurotypical controls such that their age and sex differences were minimized.
Blinding	We randomly split participants into train/test/validation splits to evaluate cross-validation classification accuracy. The test dataset was blinded from us during model training.

Reporting for specific materials, systems and methods

We require information from authors about some types of materials, experimental systems and methods used in many studies. Here, indicate whether each material, system or method listed is relevant to your study. If you are not sure if a list item applies to your research, read the appropriate section before selecting a response.

Materials & experimental systems

n/a	Involved in the study
☒	☐ Antibodies
☒	☐ Eukaryotic cell lines
☒	☐ Palaeontology and archaeology
☒	☐ Animals and other organisms
☒	☐ Clinical data
☒	☐ Dual use research of concern
☒	☐ Plants

Methods

n/a	Involved in the study
☒	☐ ChIP-seq
☒	☐ Flow cytometry
☒	☐ MRI-based neuroimaging
